# Native Word Order Processing Is Not Uniform: An ERP Study of Verb-Second Word Order

**DOI:** 10.3389/fpsyg.2022.668276

**Published:** 2022-03-30

**Authors:** Susan Sayehli, Marianne Gullberg, Aaron J. Newman, Annika Andersson

**Affiliations:** ^1^Centre for Research on Bilingualism, Stockholm University, Stockholm, Sweden; ^2^Centre for Languages and Literature, Lund University, Lund, Sweden; ^3^Department of Psychology and Neuroscience, Dalhousie University, Halifax, NS, Canada; ^4^Department of Swedish, Linnaeus University, Växjö, Sweden

**Keywords:** ERP, N400, P600, language processing, variation, word order

## Abstract

Studies of native syntactic processing often target phrase structure violations that do not occur in natural production. In contrast, this study examines how variation in basic word order is processed, looking specifically at structures traditionally labelled as violations but that do occur naturally. We examined Swedish verb-second (V2) and verb-third (V3) word order processing in adult native Swedish speakers, manipulating sentence-initial adverbials (temporal *idag* ‘today’, spatial *hemma* ‘at home’ and sentential *kanske* ‘maybe’) in acceptability judgements, in simultaneously recorded event-related potentials (ERP) to visually presented sentences and in a written sentence completion task. An initial corpus study showed that the adverbials differ in frequency in fronted position (*idag* > *kanske* > *hemma*), and although all occur mainly with V2 word order, *kanske* occurs more frequently with V3 in natural production than both *idag* and *hemma*. The experimental results reflected these patterns such that V2 sentences were overall more frequently produced and were deemed more acceptable than V3 sentences. The ERP results consisted of a biphasic N400/P600 response to V3 word order that indicated effects of word retrieval and sentence reanalysis. We also found consistent effects of adverbials. As predicted, V3 was produced more frequently and judged as more acceptable in *Kanske* sentences than in sentences with the other two adverbials. The ERP analyses showed stronger effects for *idag* and *hemma* with V3, especially regarding the P600. The results suggest that the naturally occurring word order ‘violation’, V3 with *kanske*, is processed differently than V3 with other adverbials where the V2 norm is stronger. Moreover, these patterns are related to individuals’ own production patterns. Overall, the results suggest a more varied native word order processing than previously reported.

## Introduction

Much research on language processing has focused on ambiguity resolution and on the effect of syntactic complexity on integration (for a review, MacDonald and Hsiao, 2018). Much less work has examined how variation in basic syntactic structures is handled. The current study explores the processing of basic word orders, such as the Germanic verb-second (V2) word order in which the finite verb occurs in second position in a main clause regardless of whether it starts with a subject (e.g. *she*; SVO) or an adverbial (e.g. *today*; AdvVSO). Although V2 is normally regarded as a robust word order rule, speakers of V2 languages have been observed to occasionally also produce clauses with V3 word order (AdvSVO), for example following specific sentence adverbials (e.g. *kanske hon vill låna min cykel* lit. Maybe she wants borrow my bike, Bohnacker, 2006, p. 455). However, virtually nothing is known about the real-time native processing of such basic word order variation, including what has traditionally been regarded as word order violations (but see Duffield et al., 2007). This study therefore investigated V2/V3 processing in 20 adult Swedish native speakers, manipulating sentence-initial adverbial (temporal *idag* ‘today’, spatial *hemma* ‘at home’ and sentential adverbial *kanske* ‘maybe’) in a sentence completion task, in untimed acceptability judgements, and simultaneously recorded event-related potentials (ERP) to visually presented sentences.

## Background

### V2 Word Order

In so-called verb-second or V2 languages (such as all mainland Scandinavian languages, German and Dutch), the finite verb appears in second position in main clauses, preceded by only one constituent regardless of whether it is a subject, adverbial or something else. If an element other than the subject appears in the initial position of a declarative main clause, the subject appears in third position leading to so-called *subject-verb inversion* (XVS where X can be any other constituent) or V2. In Swedish this affects approximately 40% of all declarative utterances in spoken production both in adult language and child directed speech regardless of register or dialect (Jörgensen, 1976; Håkansson, 1988; Håkansson and Nettelbladt, 1996). Most spoken XVS sentences start with a fronted adverbial; objects in first position are information structurally marked in Swedish. The most frequently fronted adverbials appear to be temporal (Jörgensen, 1976; for child directed speech, Josefsson, 2003). The proportion of XVS or V2 has been assumed to be higher in written than spoken Swedish (Westman, 1974; Jörgensen, 1976).

Although V2 is generally considered to be uniform in native Swedish, cases where the finite verb occurs in third position without subject-verb inversion (XSV or V3) are nonetheless found in native speaker production. In simple declarative main clauses, such deviations from V2 have traditionally been discussed as word order violations. However, they appear to be acceptable with certain fronted adverbials (e.g. Jörgensen, 1976; Platzack, 1998; Bohnacker, 2006; Andréasson, 2007; for a discussion of acceptable norm violations in languages, see Hubers et al., 2020). The Swedish Academy Grammar of Swedish (SAG) states that declaratives with sentential adverbials, such as *kanske*, *måhända* and *kanhända* ‘maybe, perhaps’ in first position can optionally occur with inverted (V2) or non-inverted (V3) word order (SAG; Teleman et al., 1999), as in (1a) and (1b).^1^

1.
  a. V2: *Kanske har han* […] *varit där i dag*.
                  maybe has he […] been there today
  b. V3: *Kanske han* […] *har varit där i dag*.
                  maybe he […] has been there today
    (SAG; Teleman et al., 1999, Vol. 4, p. 21–22).

Jörgensen (1976) also notes that *sedan* (‘then’) occasionally occurs with V3 word order when fronted (see also Bohnacker, 2006). Overall, however, there is little quantitative information on how common such optional V3 word orders with fronted adverbials really are in standard native Swedish, although it is often assumed that it is more frequent in spoken, informal production (cf. Ganuza, 2008). It is worth noting that V3 word order frequently occurs in non-native production of V2 languages (Hyltenstam, 1977, 1978; Bolander, 1988; Håkansson and Nettelbladt, 1996) and in so-called contemporary urban vernaculars, that is, youth speech styles originating from multi-ethnic urban areas. In most Germanic urban vernaculars, V3 word order is thought to be frequent and a distinguishing feature (Kotsinas, 1998; Quist, 2008; Wiese, 2009; Freywald et al., 2015; for an overview, Walkden, 2017). However, in a corpus of Swedish urban vernaculars, V3 word order occurred in only 7.4% of the topicalised sentences with an adverbial in first position (Ganuza, 2008).

In sum, although V2 word order in main clauses with fronted adverbials is the standard in native Swedish, some variations are clearly present in native production, notably with fronted sentential adverbial *kanske* ‘maybe’. However, Swedish basic word order has mainly been studied in offline language production. Very little is known about how Swedish basic word order is processed in online sentence comprehension.

### Word Order Processing

The literature on word order processing often examines effects on processing of violations of word order rules, but also effects of variation, as determined by ‘typicality’ or frequency. Studies commonly investigate processing costs behaviourally through longer reaction or reading times (e.g. Meng and Bader, 2000; Hopp, 2006; Duffield et al., 2007) and neurocognitively in quantitatively or qualitatively different ERP effects (for reviews, see Kaan, 2007; Hagoort and Indefrey, 2014).

In research on the processing of word order violations, neurocognitive ERP studies typically report a biphasic effect consisting of an anterior negativity, sometimes left lateralised (LAN), followed by a parietal positivity with an onset around 600 ms after critical word onset, the P600 (for a review, Swaab et al., 2012). In these studies, the negativity is described as an automatic response to the violation while the P600 is described as related to a reanalysis of the sentence or a syntactic integration difficulty (see, e.g. Van Petten and Luka, 2012). The latter interpretation is supported by the fact that the P600 is elicited also in correctly formed sentences that require reanalysis, such as in garden path sentences and syntactically more complex sentences (Osterhout and Holcomb, 1992, 1993; Osterhout et al., 1994; Osterhout, 1997; Kaan et al., 2000; Friederici, 2002; Kaan and Swaab, 2003; Brouwer et al., 2012). Importantly, however, most studies of word order violations typically do not study naturally occurring violations but rather phrase structure violations, such as *…Max’s of proof the theorem (Neville et al., 1991, p. 154). These violations elicit the biphasic response (LAN/P600) indicating processing difficulties. The biphasic response has been reported in several subsequent studies (e.g. Friederici et al., 1993, 2002; Hagoort et al., 1993; Weber-Fox and Neville, 1996; Hahne and Friederici, 2002; Isel et al., 2007; Yamada and Neville, 2007; Steinhauer et al., 2010). In offline acceptability judgement tasks, similar phrase structure violations are usually related to lower acceptance rates (Fanselow and Frisch, 2006; Häussler et al., 2015; Almor et al., 2017). In contrast, very few studies have examined the processing of naturally occurring basic word order ‘violations’, such as V3 in V2 languages.

Weyerts et al. (2002) investigated the processing of SVO (which they referred to as V2) and SOV in German main and embedded clauses. They compared ungrammatical SOV in the main clause with grammatical SOV in the embedded clause and reported an increased positivity for ungrammaticality. They also reported a frontocentral negativity for SOV regardless of grammaticality. However, when comparing SVO in both clause types they found no ERP effect related to grammaticality. They concluded that these patterns were related to a preference for or ease of processing of the more common SVO word order. A study of Dutch took a similar approach (den Ouden and Bastiaanse, 2009). This ERP study showed a sustained anterior negativity that did not differ with word order and that could be an indication of an increased working memory load. Similarly to Weyerts et al. (2002), the study also found a P600, but in this case both for SOV in main clauses and for SVO in the embedded clause, thus in contrast to the previous study restricted to contexts of word order violations.

Importantly, in the first of these studies (Weyerts et al., 2002) the ERPs were time-locked to V and O, respectively. It is therefore not clear whether the different effects were related to a different processing of verbs and nouns or to word order *per se* (for a more thorough discussion, see Schlesewsky et al., 2002; den Ouden and Bastiaanse, 2009). However, regardless, the null effect of SVO in embedded clauses replicated the null effect in their first experiment, a self-paced reading study where ungrammatical VO was read as quickly as grammatical OV, while ungrammatical OV in the main clause differed from grammatical VO. Also, it is unclear for the parser in both studies (Weyerts et al., 2002; den Ouden and Bastiaanse, 2009) that SVO is incorrect until after it has processed the V—it is only then that the transitive structure becomes clear and that the word order is revealed as incorrect. In contrast to these two studies, a recent study used S as the critical word, since the V2 word order was investigated in sentences starting with an adverbial phrase that should, according to normative syntax, be followed by a V. Violations of V2 word order in sentences with long prefields (i.e. a long first constituent consisting of several adverbials, e.g. *idag efter skolan* ‘today after school’), elicited a biphasic response (LAN/P600; Andersson et al., 2019).

In contrast to studies of violations of word order, a different line of research targets effects of *variation* in basic word order. These studies often suggest frequency effects such that frequent structures are preferred or more easily processed than less frequent ones (e.g. Friederici et al., 1998, 2002; Rösler et al., 1998; Vos et al., 2001; Fiebach et al., 2002; but see Yamashita, 1997; Mishra et al., 2011). For example, the first noun phrase of a main or subordinate clause is often interpreted as a subject rather than an object (the subject-first preference; MacWhinney et al., 1984; Frazier, 1987; Schriefers et al., 1995; Hyönä and Hujanen, 1997; Kaan, 1997), even when other constituents can occur sentence initially (e.g. Basque; Erdocia et al., 2009). For instance, in Spanish, the less frequent but well-formed OVS word order elicited stronger anterior negativities in comparison to the more common SVO order (Ostrosky-Solis et al., 1996). In cases eliciting a sustained negativity this has been explained as reflecting an increased memory load, while the following positivity has been argued to reflect difficulties with syntactic integration (e.g. Fiebach et al., 2002). Importantly, the positivity found in response to syntactically more complex sentences is often more frontally distributed than the positivity typically elicited by word order violations (Friederici et al., 2002). Thus, the different distributions have been suggested to indicate either difficulties with syntactic integration (frontal distribution) or with reanalysis (posterior distribution).

The processing of preferred word order has also been studied in Swedish. Hörberg et al. (2013) investigated the processing of information structurally marked sentences with the object in the first position (OVS vs. SVO), focusing on the effects of grammatical functions and pronominal case marking on syntactic reanalysis. They reported what they referred to as a ‘reanalysis N400’, that is, a negativity in the N400 time window over right parietal sites when the parser reached the subject pronoun in nominative case and realised that the sentence-initial noun was not the subject. Dröge et al. (2016) also investigated preferred and non-preferred word orders, SO and OS following the verb in answers to questions where subjects and objects were case marked (e.g. *Ja natürlich verfolgt der Nachtwächter den Dieb,* lit. ‘Yes of course chases the.SUBJ night guard the.OBJ thief’ vs. *Ja natürlich verfolgt den Dieb der Nachtwächter*, lit. ‘Yes of course chases the.OBJ thief the.SUBJ night guard’). They reported a biphasic response where the negativity was elicited by OS regardless of whether case marking or animacy indicated the syntactic role, while the subsequent positivity was stronger with case marking. That is, when there was no ambiguity of syntactic role and the parser could be certain of the mismatch of expectation, the positivity was stronger.

Another negativity is of interest for the current study, the N400. This centro-medial negativity with a peak around 400 ms post critical word onset has traditionally been discussed as indicating semantic integration (Kutas and Hillyard, 1980, 1984; Holcomb and Neville, 1991) or retrieval of lexical–semantic information (Kutas and Federmeier, 2000; Lau et al., 2008; Brouwer et al., 2017). The effect decreases with expectancy, such that each word in a sentence elicits the N400 and the amplitude is reduced with each word in the sentence as the words concur with the expectation (Kutas and Hillyard, 1980). Similarly, the negativity reported for non-preferred word order is thought to indicate a violation of expectation of a particular word or lexical item (e.g. Dröge et al., 2016; Kuperberg et al., 2020). Such effects of violations of expectancy have been successfully computationally modelled (Brouwer et al., 2021). The computational model combined a neurocomputational model (Brouwer et al., 2017) with a comprehension model (Venhuizen et al., 2019) and took each subsequent word and previous linguistic experience into account for prediction of the upcoming word. Difficulties of processing were negatively related to expectancy and positively to amplitude of the ERP effects (N400 and P600). These combined results indicate that the N400 indexes retrieval processes and are therefore sensitive to expectancy. Importantly, neither the N400 nor the P600 should be thought of as related to a linguistic domain (semantics, pragmatics or syntax) but rather to cognitive processes related to expectancy and surprisal (see Dröge et al., 2016).

In support of such interpretations, studies investigating second language processing have reported differences in native and non-native processing such that syntactic processing is reflected both in a P600 and a N400 (Osterhout et al., 2006; Mueller et al., 2009; Carrasco-Ortíz et al., 2017; Kimppa et al., 2019). These differences in elicited ERP effects to syntactic violations are believed to represent the fact that word retrieval is comparably undemanding for native speakers, such that the ERP response is restricted to a P600 (Brouwer et al., 2017; Brouwer and Crocker, 2017). In contrast, the N400 effect in non-native speakers has been suggested to indicate a reliance on lexical or semantic processes for processing morphosyntax (e.g. Kimppa et al., 2019). Importantly, such individual differences in elicited effects (N400 or P600) have also been reported in native syntactic processing (Osterhout, 1997), suggesting that changes to processing demands yield different ERP effects. Accordingly, if the task is demanding or proficiency in the language is low, syntactic processing of violations can be reflected in an increased N400 rather than an increased P600. However, these individual differences in effects can show up as a biphasic response in grand average waveforms (McLaughlin et al., 2010; Tanner et al., 2014; Tanner and Hell, 2014).

In a recent paper, Fromont et al. (2020) have also suggested that a biphasic N400-P600 response can be considered to be an index of word order violations (referred to as a syntactic category violation in the paper). When controlling for the word preceding the critical word (e.g. in the French stimuli either the determiner *le* or the clitic pronoun *le*), they found an N400 effect rather than a LAN to the word order violation (substituting a verb for a noun and vice versa) followed by a P600. In addition, they manipulated a preceding context sentence so that a critical word was semantically primed or not primed which affected the N400 amplitude of the critical word, such that the effect was similar to that from the word order violation manipulation. Importantly, however, when presenting the syntactic category violation simultaneously with the semantic violation, they reported additive effects, suggesting that the N400 effect for the two types of violations are distinct.

In sum, previous behavioural and neurocognitive work on the processing of word order has either targeted (phrase structure) violations or frequency effects of naturally occurring word order variation, such as SVO and OVS orders. Both violations and low frequencies seem to incur lower acceptance rates and higher processing costs. However, so far little is known about the online processing of optionally occurring variations in main clauses traditionally regarded as basic word order violations. In the current study, we therefore probe the processing of ‘correct’ V2 and ‘incorrect’ V3 structures, traditionally regarded as violations, but that occur in native speaker production, possibly at lower frequency rates.

## Current Study

This study sets out to examine whether native speakers of Swedish are sensitive to V2-V3 variations in basic word order and specifically whether effects differ depending on the single, fronted adverbial and its frequency. According to older corpus studies temporal adverbials are more often fronted in Swedish than spatial adverbials (Jörgensen, 1976), but the exact frequencies of fronted temporal, spatial and sentential adverbials are not known, nor to what extent they allow for V2 and V3 word orders, respectively. We therefore first carried out a corpus study to examine the frequencies of fronted temporal (*idag* ‘today’), spatial (*hemma* ‘at home’) and sentential (*kanske* ‘maybe’) adverbials in V2 and V3 sentences in contemporary Swedish. We then tested Swedish native speakers on an untimed acceptability judgement task after each visually presented sentence during which event-related potentials (ERP) were recorded and their performance on a sentence completion task.

### Preamble—A Corpus Study

Using the online search tool KORP available at Språkbanken (Borin et al., 2012; Språkbanken, 2018), we searched for V2 and V3 word order structures in the collection of corpora referred to as Bloggmix. Bloggmix consists of 615,658,549 tokens (39,171,429 sentences) gathered from 1998 onwards and represents a good sample of informal written contemporary Swedish. In preparation for the experiments with a focus on word orders following the temporal adverbial *idag*, 'today', the spatial adverbial *hemma*, 'at home' or the sentential adverbial *kanske* ‘maybe’, we searched Bloggmix for V2 (AdvVS) and V3 (AdvSV) word orders in main clauses following these three adverbials. Excluded were instances of these adverbials followed by further adverbial modifiers (underlined for presentation purposes), such as *idag mellan 14 och 21* ‘today between 2 and 9 pm’ or instances of stacking of the relevant adverbials as in *idag kanske vi har tur* ‘today maybe we’ll be lucky’. The sample is thus narrowly and strictly defined and constitutes a very small sub-sample of the entire corpus. Table 1 presents the incidence of V2 vs. V3 word order in Bloggmix.

**Table 1:** Incidence (raw frequencies) of V2/V3 word order in Bloggmix following sentence-initial adverbials.

Adverbial	V2 word order	V3 word order
*Idag* ‘today’	6,158	25
*Hemma* ‘at home’	46	7
*Kanske* ‘maybe’	1,184	243
Total	7,388	275

The corpus data clearly indicate that V2 word order dominates overall, especially following the temporal adverbial *idag* ‘today’. However, the incidence of V3 word order is not negligible in the corpus, especially following the sentential adverbial *kanske* ‘maybe’. Log likelihood tests (Rayson, 2018), which allow for comparisons across corpora of different sizes (*cf.* Rayson and Garside, 2000), reveal that V3 word order is significantly more frequent with the sentential adverbial *kanske* ‘maybe’ than with the time adverbial *idag* ‘today’ (*LL* = 900.96, *p* < 0.001) and the spatial adverbial *hemma* ‘at home’ (*LL* = 5.74, *p* < 0.05). V3 word order is also significantly more frequent with *idag* ‘today’ than with *hemma* ‘at home’ (*LL* = 34.19, *p* < 0.001), although this difference is hardly meaningful given the small numbers. Examples of V2 word order with *kanske* in the corpus include *kanske tar folk bussen* lit. Maybe catch people the buss, ‘maybe people catch the bus’, *kanske är det OK* lit. Maybe is it OK, ‘maybe it is OK’. Conversely, examples of V3 word order with *kanske* include *kanske förkylningen ger sig* ‘maybe the cold disappears’, *kanske man ska sluta* ‘maybe one should stop’.

The corpus analysis thus supports previous observations from spoken corpora (Jörgensen, 1976; Josefsson, 2003) to the effect that the V2 pattern is overall strong in Swedish main clauses with sentence-initial temporal and (to some extent) spatial adverbials and that V3 word order is relatively more common with the sentential adverbial *kanske* ‘maybe’ in (written) production. Moreover, the spatial adverbial *hemma* ‘at home’ rarely occurs sentence initially regardless of word order.

### Predictions

Based on previous findings we expect to find overall effects of V3 word order in sentence completion, acceptability judgements and ERP effects. We expect V2 to be more frequent than V3 in the sentence completion task and V2 word order to be more acceptable in the acceptability judgements. Neurocognitively, we expect an N400 to be followed by a P600 indicating difficulties with integration of the presented word category into the particular context. The N400 effect of word order violation is predicted rather than an anterior negativity since the early presentation of the critical word in the sentence does not allow for a build-up of expectancy. Instead, the N400 would be an indication of difficulties with word retrieval (e.g. Choudhary et al., 2009; Brouwer et al., 2017) in the cases where the presented word is not an expected verb (see Fromont et al., 2020 for a biphasic N400/P600 response to word category violations).

We also predict effects for the individual sentence-initial adverbials. In the sentence completion task, we expect a higher rate of V3 word order with *kanske* in comparison to sentences initiated with *hemma* and *idag*. Similarly, we expect higher acceptability for V3 word order with *kanske* than with *idag* and *hemma*. These patterns are expected to be reflected in differences in the ERP effects, such that the effects with the temporal and the spatial adverbial are expected to be similar in amplitude, while the amplitude of the effects with the sentential adverbial is expected to be attenuated since both V2 and V3 occur naturally, thus replicating previous studies showing an attenuated P600 with more uncertainty (Dröge et al., 2016).

## Materials and Methods

### Participants

A total of 20 native Swedish speakers were recruited at Lund University (excluding students of linguistics). They filled in a language background questionnaire (Gullberg and Indefrey, 2003) and a socioeconomic status (SES) questionnaire (Hollingshead, 1975). All participants had normal or corrected to normal vision, reported normal hearing, were right-handed according to the Edinburgh handedness questionnaire (Oldfield, 1971) and had no history of neurological or language disorders (results from the language background and SES questionnaires were used for a different study and will not be reported on further here). Participant characteristics are summarised in Table 2.

**Table 2:** Participants.

*N* (Females)	Age (*SD*)	SES (*SD*)
20 (8)	23;10 (4;9)	48 (14.7)

Age, given in Years; Months. SES, socioeconomic status according to Hollingshead (1975, range 0-66).

### Tasks and Materials

#### Sentence Completion Task

**Figure 1:**
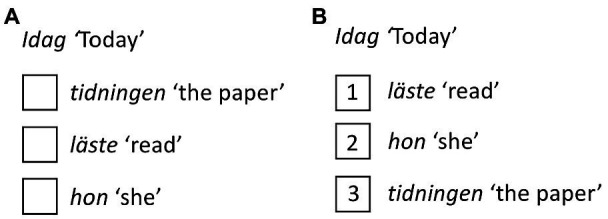
Sentence completion task (SCT). Sentences were presented with the adverbial on top followed by words or phrases below **(A)**. By inserting a number in the empty boxes, the order of the words or phrases shifted as illustrated in **(B)**. See Supplementary Materials for a full list of stimuli sentences.

A computer-based sentence completion task (SCT) was developed to test participants’ (written) production of word order. Each trial consisted of a lead-in fragment followed by boxes with words or word combinations which had to be ranked from 1 to 3 so that the sentence could be read from top to bottom (Figure 1).

In the experimental sentences (60), the lead-in fragment consisted of one of three adverbials, the temporal adverbial *idag,* 'today' (15), the spatial adverbial *hemma,* 'at home' (15) or the sentential adverbial *kanske* ‘maybe’ (30). Grammatical subjects and verbs had to be ordered as described above. Grammatical subjects consisted of lexical singular nouns (*flickan,* 'the girl', *pojken* ‘the boy’) and personal pronouns in third person singular (*hon,* 'she', *han* ‘he’) equally distributed across the three adverbials. Verbs occurred in the simple past and were selected from the most frequent verbs in the Parole corpus (2012); range: 10–4,743, *M* = 887, *SD* = 1006.43. The experimental sentences were intermingled with fillers (180). These consisted of four sentence types, namely, wh-questions, adverbial-first, subject-first and negated subject-first sentences. Filler sentences contained 15 additional sentences starting with *idag* and 15 with *hemma* with long lead-ins that were analysed for a previous study (Andersson et al., 2019), to the effect that participants constructed sentences equally often with all three adverbials *idag*, 'today', *hemma*, 'at home' and *kanske* ‘maybe’. Filler sentences also contained different adverbials from the experimental items but were matched for type: one temporal adverbial (*igår* 'yesterday'), one spatial adverbial (*här* 'here') and three sentential adverbials (*förmodligen* 'probably'*, självklart* 'obviously' and *naturligtvis* ‘of course’).

A total of 240 sentences (see Supplementary Materials) were presented to each participant. Sentences were pseudo randomised with the constraint that no more than three sentences of the same type could appear in a sequence.

#### Acceptability Judgement Task

To probe comprehension offline, we administered an untimed acceptability judgement task (AJT) while recording ERPs. The forced choice task followed each sentence where participants had to press either a green or red button to indicate whether the sentence was ‘good’ or ‘not so good’ (counterbalancing response hand for green or red across participants; see below for presentation details under the section on ERP recordings).

We presented grammatical sentences with V2 (120) and ungrammatical sentences with V3 word order (120), varying the adverbial as in the SCT. The verbs and grammatical subjects were identical to those that participants subsequently used in the SCT. To control for ERP wrap-up effects following the final word of the sentence (Osterhout and Holcomb, 1992, 1993; Hagoort et al., 1993; Osterhout and Nicol, 1999), a final phrase differing in word length was added (0–5 words; see Supplementary Materials). The experimental sentences were intermingled with fillers (240), that consisted of V2/V3 sentences with multi-word prefields with initial *idag* ‘today’, *hemma* ‘at home’ or a further adverbial *naturligtvis* ‘of course’, yielding a total of 480 sentences presented to each participant. Two lists were created counterbalancing the distribution of sentences as V2 or V3, such that each participant read an item either as a V2 or a V3 sentence (Table 3).

**Table 3:** Example and number of items of the experimental sentences in the AJT/ERP recordings.

V2	#Items	V3	#items
*Idag spelade hon/flickan piano* ’Today played she/the girl piano’	40	*Idag hon/flickan spelade piano* ’Today she/the girl played piano’	40
*Hemma spelade hon/flickan piano* ’At home played she/the girl piano’	40	*Hemma hon/flickan spelade piano* ’At home she/the girl played piano’	40
*Kanske spelade hon/flickan piano* ‘Maybe played she/the girl piano’	40	*Kanske hon/flickan spelade piano* ‘Maybe she/the girl played piano’	40
	120		120

#### ERP Recordings

Sentences were visually presented to participants word by word (white Arial, 22 pt. on black background) in the centre of a computer screen 130 cm in front of the participant while the electroencephalogram (EEG) was recorded and time-locked to the critical word (the grammatical subject), the first word at which the word order violation first could be detected. Words were presented for 300 ms with an inter-stimulus interval (ISI) of 200 ms to reduce early ERP effects related to the word preceding the critical word (Steinhauer and Drury, 2012). Sentence-final words were followed by full stops. No other punctuation was included. The final word was followed by a blank screen for 700 ms. Three question marks then appeared on the screen until the acceptability judgement was made. The timing of the judgement (reaction time) is measured by E-prime (Schneider et al., 2012) by default. A subsequent button press moved the experiment on to the next test item. The EEG was recorded from 29 electrodes mounted in an elastic cap (EASYCAP). Data from 12 pairs of lateral sites (F7/8, FT7/8, F3/4, FC3/4, T7/8, TP7/8, C3/4, CP3/4, P7/8, P3/4, PO7/8 and O1/2) were included in analyses while FP1/2 and the three midline sites (FZ, CZ and PZ) were only used for detecting artefacts. This was also the case for the four additional electrodes that monitored blinks (above and below the left eye, i.e. VEOG) and eye movements (at the outer canthi of both eyes, i.e. HEOG). These electrodes had an impedance maintained below 10 kΩ while the impedance of all other electrodes was maintained below 5 kΩ. Neuroscan SynAmps2 (bandpass 0.05–0.100 Hz) was used to amplify the EEG that was digitised at a sampling rate of 500 Hz. Each scalp electrode was referenced to Cz during recording and re-referenced to the averaged mastoids during offline processing.

### Procedure

Participants signed consent forms in accordance with the Declaration of Helsinki and then filled in the language background, handedness and SES questionnaires. The experimental session started with the recording of ERPs and the AJT. Following the ERP session, participants took the SCT, a Swedish proficiency test (Swedex, 2012) and an English proficiency test (the Oxford placement test 2; Allen, 1992). The proficiency tests were administered because the data collection was part of a bigger study which also involved non-native speakers of Swedish as a second language (cf. Andersson et al., 2019). A complete session typically lasted for 2 h. Participants were debriefed after the session and awarded two movie tickets for their participation.

### Data Treatment and Analyses

#### Sentence Completion Task and Acceptability Judgement Task

A generalised linear mixed model (IBM SPSS Statistics 26) was used to analyse responses to the sentence completion task (SCT) to estimate the variance in the binary outcome variable (V2 vs. V3) with adverbial (temporal/spatial/sentential) as the predictor variable and items and participants as random effects considering the repeated measures.

For the AJT data, d-prime (*d’*) scores (Wickens, 2002) were computed to measure response accuracy such that *d’* = 4 suggested near-perfect discrimination between V2 and V3 word orders and *d’* = 0 chance level performance. The *d’* scores were normally distributed as assessed by a Shapiro–Wilk test (*p* > 0.05). A one-way repeated measure analysis of variance (ANOVA) was conducted to test the effects of the within-subject factor (adverbial: temporal/spatial/sentential) on the *d’* scores. Since the Mauchly’s test of sphericity indicated that the data violated assumptions of sphericity [*χ*^2^(2) = 50.90, *p* < 0.001], Greenhouse–Geisser corrections were applied. In addition, Bonferroni corrections of the alpha levels were applied to *post hoc* pairwise comparisons when main effects were significant. In tables and in the body of the text only significant effects and corrected *p*-values and uncorrected degrees of freedom are reported.

#### ERP Preprocessing

All of the code used to perform the preprocessing and analysis presented here, as well as the raw data, are available on osf.io.^2^

All data preprocessing after data collection was performed using the MNE-Python software (Gramfort et al., 2013) v. 0.23.0 in Python v 3.9.6. The raw, continuous EEG data for each subject was bandpass-filtered in two ways: once using a highpass cut-off of 1 Hz (for artefact identification with independent components analysis—ICA—see below) and once with a highpass cut-off of 0.1 Hz (for further analysis). In both cases, the lowpass cut-off was 30 Hz and a zero-phase hamming-windowed finite impulse response filter was used, with MNE’s default parameters. ICA was then applied to the continuous, 1–30 Hz bandpass-filtered data, using the *fastica* algorithm (Hyvärinen, 1999) and set to produce the number of components that explained 99% of the variance in the data. MNE’s *find_bads_eog* algorithm was used to automatically identify ICA components associated with ocular artefacts (as indicated by high correlations with ocular channels), including blinks and eye movements., using a threshold of *z* > 3. ICA components meeting this criterion were removed to correct for ocular artefacts. The results of this process for each participant were inspected by an experienced EEG researcher (AJN), who confirmed that all ICA components automatically removed by this process were consistent with the known properties of such artefacts (e.g. having characteristic scalp topography, timing and frequency).

The ICA decomposition was then applied to the 0.1–30 Hz bandpass-filtered data, and the ocular components removed, for further processing and analysis. The ICA-corrected data were then segmented around the onsets of the critical words (grammatical subjects of sentences), from 1,000 ms prior to the onset of the critical word, to 1,000 ms after. The data were not baseline-corrected, but were DC offset-corrected by subtracting the mean amplitude across the whole −1,000–1,000 ms epoch, at the level of individual trials and channels. Data were then re-referenced to the average of the left and right mastoid electrodes.

As noted above, we did not baseline correct the ERP data in the conventional sense of subtracting the mean amplitude over a fixed prestimulus period from the post-stimulus data. Rather, each trial was mean-centred, and then, we applied baseline regression using the approach described by Alday (2019). In this approach, mean amplitude over a prestimulus baseline segment (in our case, −100–0 ms relative to word onset) is computed, but it is included as a variable in a regression analysis of post-stimulus data, along with the experimental variables, rather than simply being subtracted. This approach allows the baseline term to interact with experimentally manipulated variables and thus account for potential differences in the baseline (including scalp topography differences) between conditions. In the present data, this was essential because the critical words in the V2 and V3 sentence structures were, by definition, preceded by different grammatical categories of words. Such systematic prestimulus variation in words during the ‘baseline’ period have been shown to lead to erroneous interpretations of ERP data, including the appearance of post-stimulus differences in ERP amplitude that were actually induced by subtraction of very different baselines between conditions (see, e.g. Steinhauer and Drury, 2012).

The baseline regression was conducted in MNE-Python using code adapted from that published as supplementary material to Alday (2019; https://osf.io/pnaku/), including extending the code to apply baseline regression separately for each channel. For each subject, and across each trial, channel and time point, linear regression was applied with fixed effects of condition (6 levels, corresponding to each combination of sentence position and adverbial) and baseline and the interaction of those two variables. The results of this, with baseline effectively ‘regressed out’, were saved and used for visualisations as ERP waveforms and scalp topographies.

#### ERP Analyses: Statistical Analyses

For statistical analyses, we computed mean amplitudes over a set of *a priori* time windows covering the entire post-stimulus period: 100–300, 300–500, 500–700, 700–900 and 900–1,000 ms. This was done individually for each participant, trial and channel. We imported these data into the R software package v 4.1.1 (R Core Team, 2021) and performed linear mixed effects modelling using the *bam* function in the *mgcv* package v 1.8–36 (Wood, 2017).^3^ A number of candidate models were fit for each time window and compared using the Akaike information criterion (AIC; Akaike, 1973), which estimates prediction error of a model, considering both the goodness of fit and the model’s complexity. The best model for every time window included fixed effects of adverbial (*kanske*/*hemma*/*idag*), sentence position (V2/V3), region of interest (electrodes were grouped into 9 regions according to a grid of left/midline/right and anterior/central/posterior) and baseline, and all possible interactions between these variables, as well as random intercepts for subjects and for sentences.^4^ In all analyses, we ignored the interaction terms involving the baseline variable, meaning the effects of baseline were controlled for in the results, but not explicitly examined (Alday, 2019). Significant interactions involving Adverbial and Sentence Position (and, if present, ROI) were further investigated in planned contrasts of V3-V2 for each adverbial at each ROI. The *p*-values for these contrasts were corrected for the number of multiple comparisons (3 adverbials × 9 ROIs = 27 contrasts) using the false discovery rate method of Benjamini and Hochberg (1995).

## Results

### Sentence Completion Task, Acceptability Judgement Task

The results on the SCT and the AJT are presented in Table 4. In the SCT, participants produced almost only V2 sentences (*M* = 95.4, *SD* = 0.04). A generalised linear mixed model analysis suggested that sentences starting with the sentential adverbial *kanske* were less often produced with V2 word order than sentences with initial temporal (*idag*) and spatial (*hemma*) adverbials (*kanske-idag*: *Est*. = −1.37, *SE* = 0.48, *t* = −2.87, *p* < 0.05; *kanske-hemma*: *Est.* = −1.78, *SE* = 0.58, *t* = −3.19, *p* < 0.05). A one-way repeated measures ANOVA revealed a main effect of adverbial regardless of whether an outlier (*d*’ scores more than two standard deviations below the mean) was excluded [*F*(2, 38) = 28.11, *p* < 0.001, *η*^2^ = 0.61] or not [*F*(2, 38) = 29.32, *p* < 0.001, *η*^2^ = 0.61]. Table 4 presents results from all 20 participants. Pairwise comparisons showed that participants were significantly better at discriminating V2 and V3 word order in sentences with the adverbial *idag* than sentences with both *hemma* and *kanske*, which also differed from each other in that participants were better at discriminating word orders in sentences with *hemma* than with *kanske*.

**Table 4:** Scores on sentence completions and accuracy judgements.

	SCT (*SD*)	AJT (*SD*)
Idag	0.99	3.33
	(0.03)	(0.79)
Hemma	0.98	3.00
	(0.04)	(0.88)
Kanske	0.93	1.38
	(0.08)	(1.06)

SCT, sentence completion task; AJT, acceptability judgement task; SD, standard deviation. Sentence completion: proportion V2 sentences. Acceptability judgements in d-prime scores. ^**^p < 0.01.

### ERP Results

**Figure 2:**
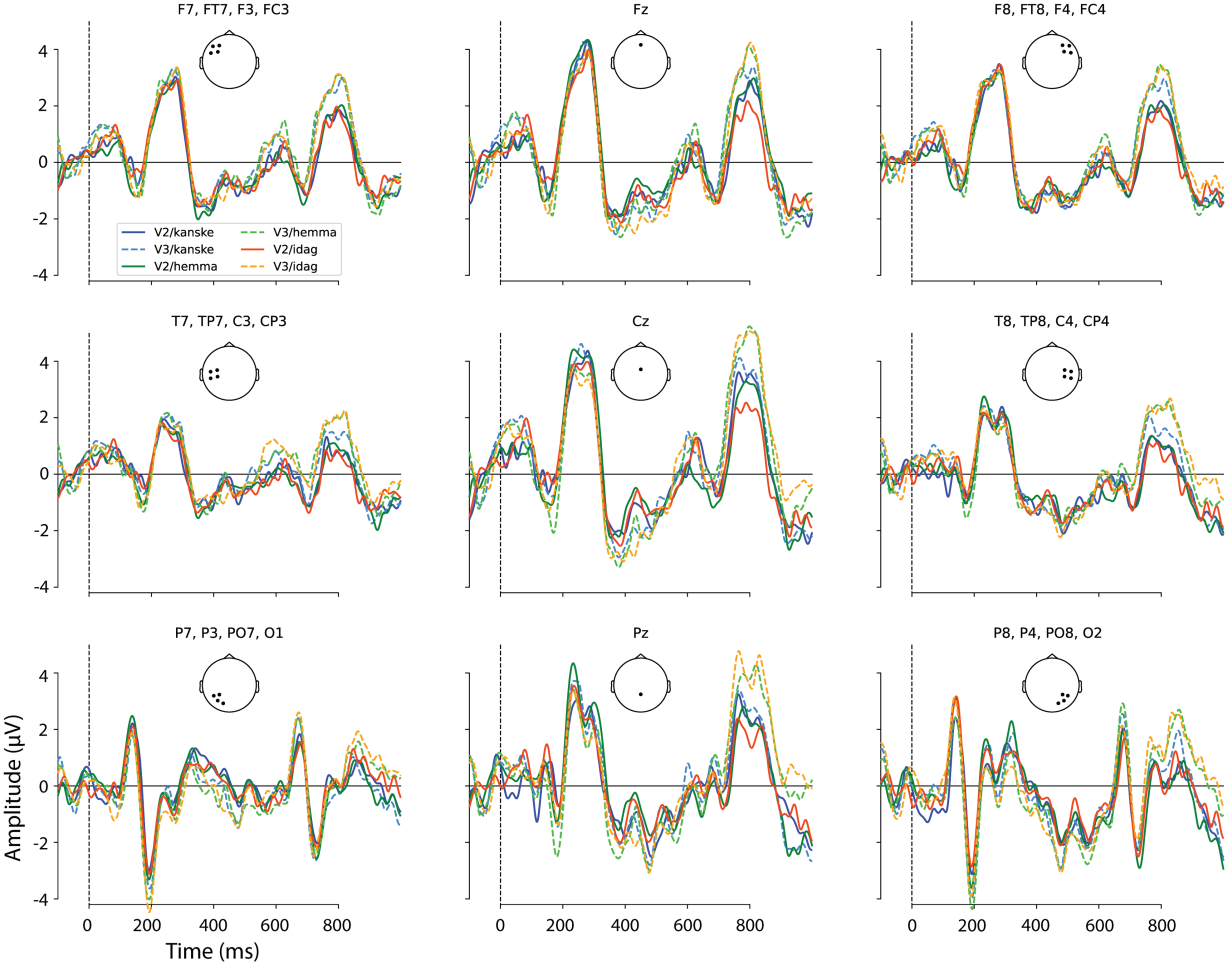
Grand average waveforms, the ERPs to V2 word order (full lines) and V3 word order (dotted lines) for each adverbial at the nine ROIs. ERPs to subjects in V2 and V3 following *hemma* in green, *idag* in red and *kanske* in blue. Positive plotted up.

With baseline regression applied, the ERP waveforms elicited by the critical words across all three adverbials and both sentence positions looked generally similar, with a classic P1-N1-P2 pattern at lateral posterior electrode sites, followed by a prominent negativity over the vertex and then a positive deflection (Figure 2).

**Figure 3:**
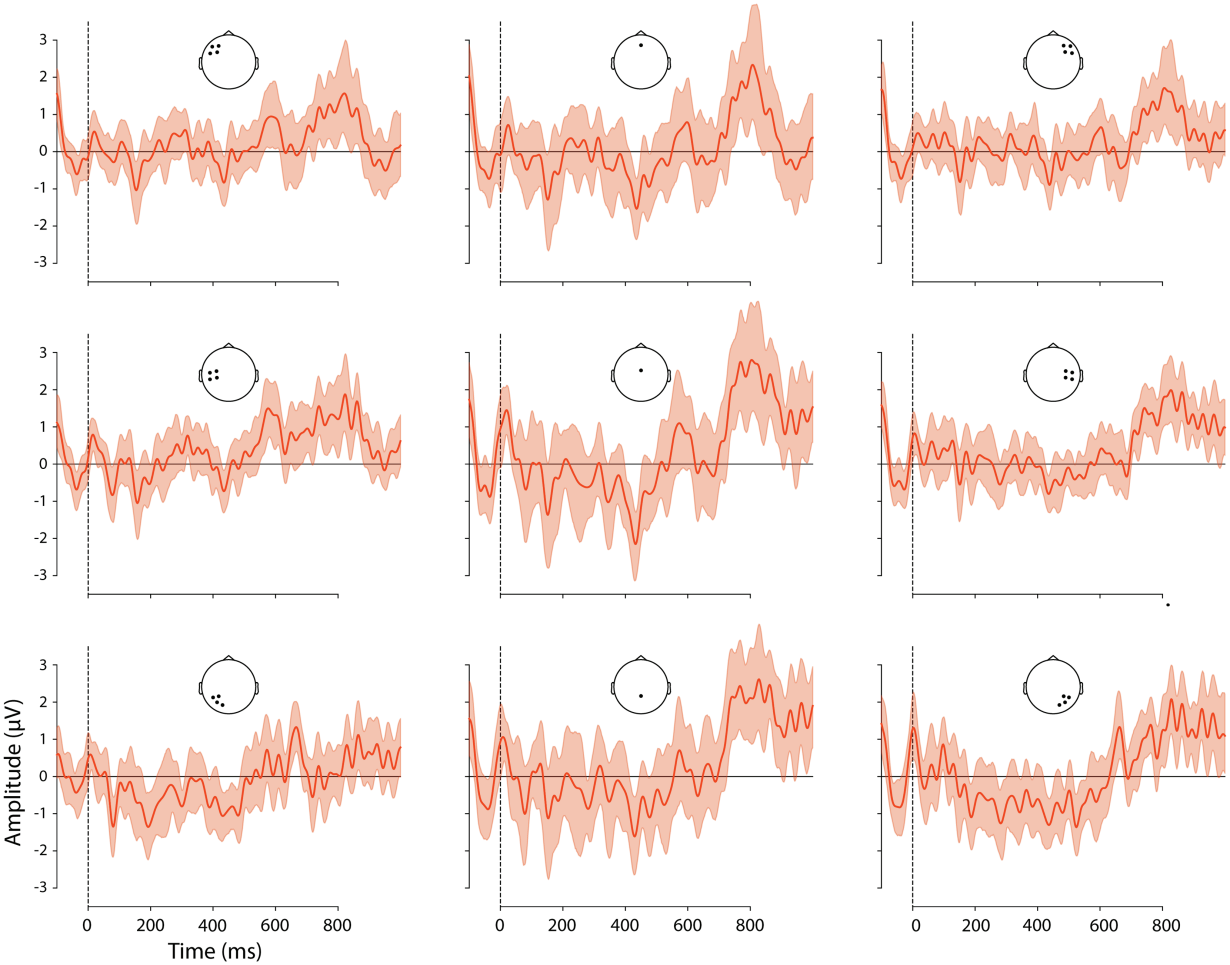
Difference waveforms, the ERPs to V2 word order subtracted from that to V3 word order following *idag* ‘today’ at the nine ROIs. Confidence intervals (95%) are shaded. Positive plotted up.

**Figure 4:**
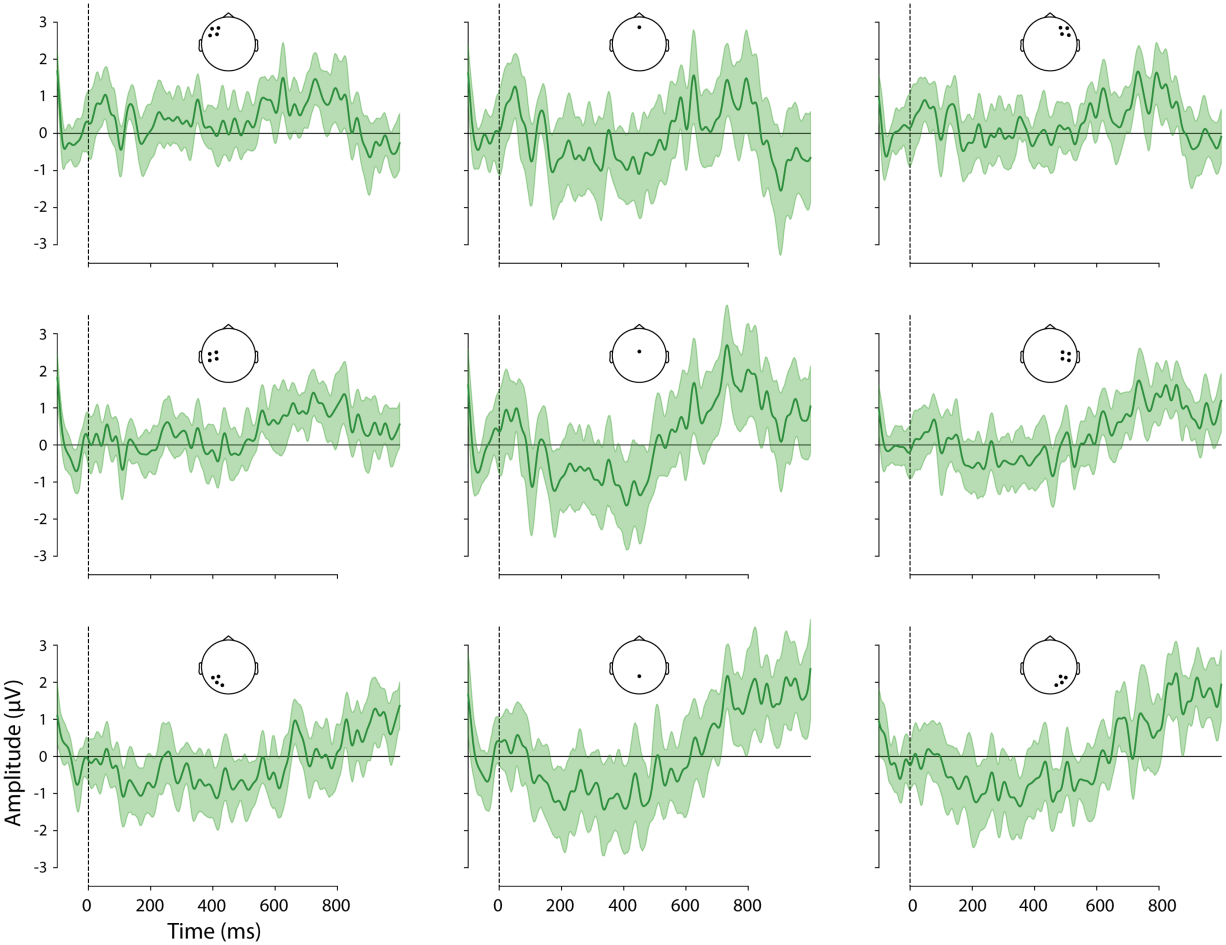
Difference waveforms, the ERPs to V2 word order subtracted from that to V3 word order following *hemma* ‘at home’ at the nine ROIs. Confidence intervals are shaded. Positive plotted up.

**Figure 5:**
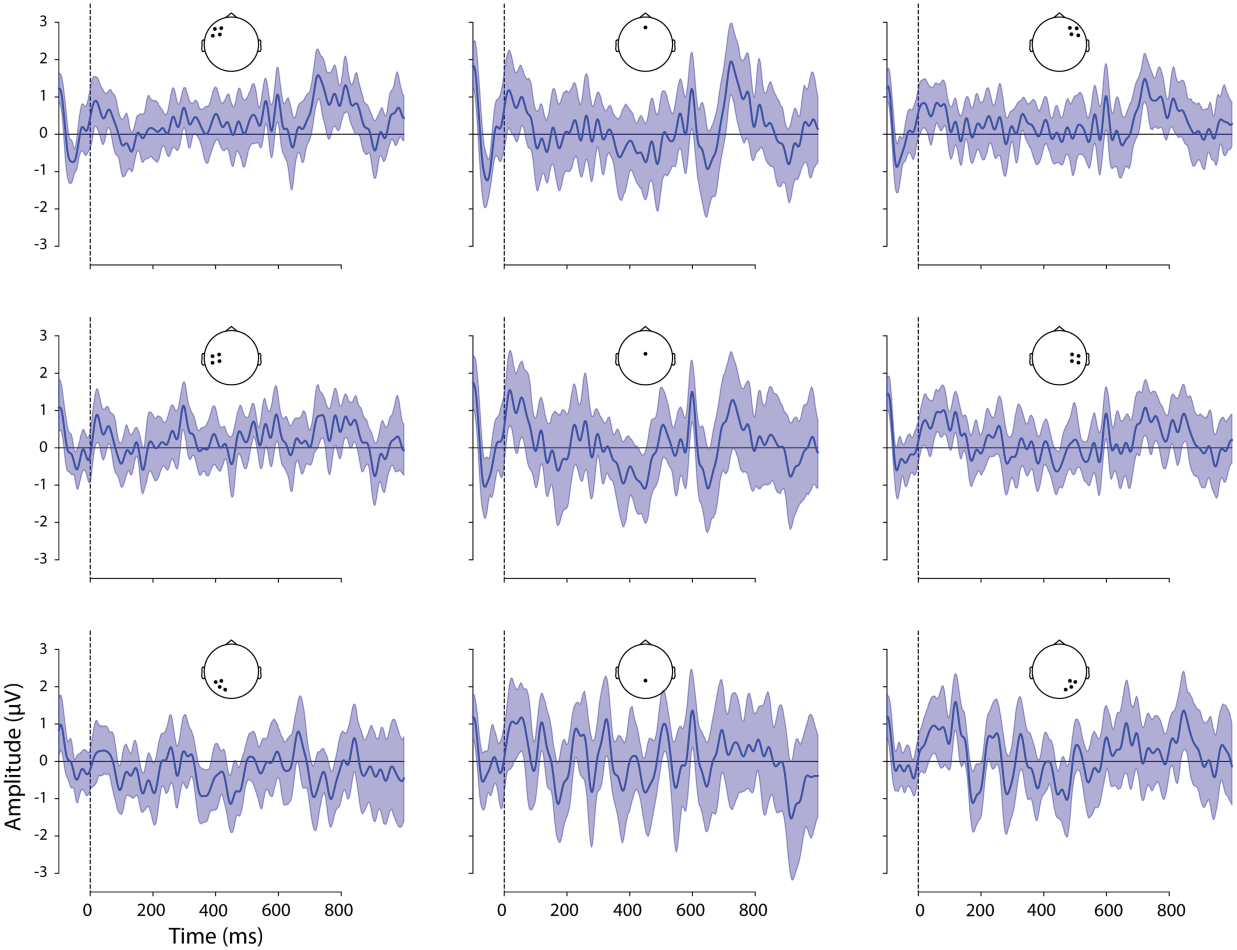
Difference waveforms, the ERPs to V2 word order subtracted from that to V3 word order following *kanske* ‘maybe’ at the nine ROIs. Confidence intervals are shaded. Positive plotted up.

**Figure 6:**
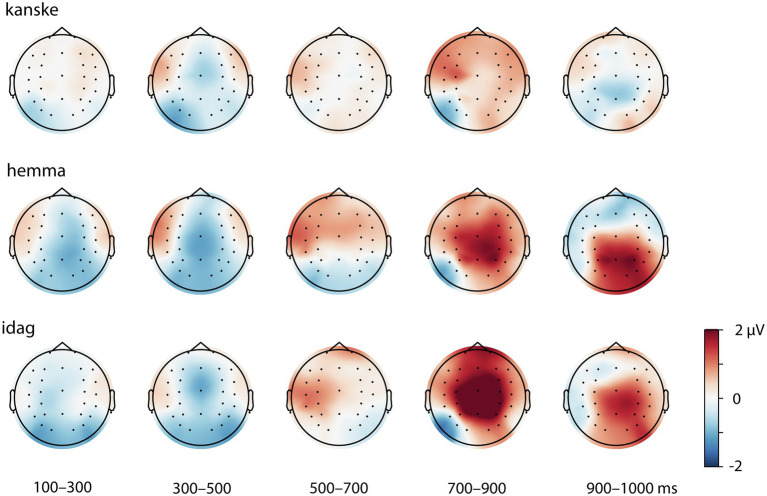
Topographic plots derived from difference waveforms. That is, the ERPs to V2 word order subtracted from that to V3 word order following each of the adverbials presented in the analysed time windows (100–300, 300–500, 500–700, 700–900 and 900–1,000 ms). Negativities in blue and positivities in amber.

Given our hypotheses regarding ERP differences between V2 and V3 sentence positions and the interaction of this factor with adverbial, we examined the difference waves created by the subtraction V3-V2, for each adverbial. These are shown, with 95% confidence intervals (CIs), in Figures 3–5 and corresponding scalp topographic maps in Figure 6. These figures suggest a negativity over the vertex and extending to posterior sites from approximately 100–500 ms—with a peak around 400–500 ms, consistent with the N400—that was most pronounced for *hemma* and *idag* and smaller for *kanske*. Notably, the CIs for the differences in this time window for *hemma* and *idag* do not include zero within parts of the N400 time window.

The N400-like negativity was followed by a greater positivity for V3 than V2 sentence positions, for all adverbials. This late positivity was maximal from 700 to 900 ms over the vertex and was largest for *idag* and smallest for *kanske*. The CI did not include zero for a clear portion of the 700–900 ms time window for all three adverbials.

For each of the *a priori* time windows of interest, we fit a family of linear mixed effects (LME) models, as detailed in the Methods, and selected the best one. In all time windows, the best model included the 4-way interaction of Adverbial × Sentence Position × ROI × Baseline, with random intercepts for Subjects and Items (i.e. sentences). For the purposes of interpretation, we ignored any interactions involving baseline, as these were not of theoretical interest but rather included to control for baseline variation (Alday, 2019). The results of these LME models are presented in Table 5, highlighting significant interactions involving the experimentally manipulated factors Adverbial and Sentence Position and, if significant, the interaction of ROI with either or both of these factors. In each time window, we focus on the highest order interaction among these three variables, as the interpretation of any lower-order interaction involving the variables would necessarily be influenced by their participation in a higher-order interaction. Note that in reporting *F* statistics, we provide the numerator degrees of freedom only. Computation of denominator degrees of freedom in LME models is a contentious topic, but in the present data set, with data treated at the level of individual trials, the denominator degrees of freedom were all >125,000, rendering small differences between computational methods functionally irrelevant.

**Table 5:** Results of the LME model for Adverbial, Sentence Position, ROI and baseline.

	*df*	*F*	*p*-value
**100–300 ms**			
Adverbial	2	0.39	0.675
SentPos	1	0.82	0.364
ROI	8	84.67	< 0.001
baseline	1	51.34	< 0.001
Adverbial × SentPos	2	1.16	0.312
Adverbial × ROI	16	2.47	0.001
Adverbial × baseline	2	0.21	0.810
SentPos × ROI	8	2.50	0.010
SentPos × baseline	1	4.89	0.027
ROI × baseline	8	3.04	0.002
Adverbial × SentPos × ROI	16	1.70	0.039
Adverbial × SentPos × baseline	2	0.30	0.740
Adverbial × ROI × baseline	16	1.86	0.019
SentPos × ROI × baseline	8	0.94	0.485
Adverbial × SentPos × ROI × baseline	16	1.67	0.045
**300–500 ms**			
Adverbial	2	0.13	0.880
SentPos	1	5.41	0.020
ROI	8	21.94	< 0.001
baseline	1	14.21	< 0.001
Adverbial × SentPos	2	3.35	0.035
Adverbial × ROI	16	0.59	0.891
Adverbial × baseline	2	1.82	0.163
SentPos × ROI	8	8.25	< 0.001
SentPos × baseline	1	5.18	0.023
ROI × baseline	8	3.40	0.001
Adverbial × SentPos × ROI	16	0.84	0.641
Adverbial × SentPos × baseline	2	3.18	0.042
Adverbial × ROI × baseline	16	2.39	0.001
SentPos × ROI × baseline	8	3.85	< 0.001
Adverbial × SentPos × ROI × baseline	16	2.46	0.001
**500–700 ms**			
Adverbial	2	1.14	0.319
SentPos	1	6.15	0.013
ROI	8	14.44	< 0.001
baseline	1	59.20	< 0.001
Adverbial × SentPos	2	3.48	0.031
Adverbial × ROI	16	2.39	0.001
Adverbial × baseline	2	2.59	0.075
SentPos × ROI	8	1.38	0.199
SentPos × baseline	1	5.50	0.019
ROI × baseline	8	6.98	< 0.001
Adverbial × SentPos × ROI	16	3.01	< 0.001
Adverbial × SentPos × baseline	2	1.20	0.302
Adverbial × ROI × baseline	16	3.16	< 0.001
SentPos × ROI × baseline	8	2.91	0.003
Adverbial × SentPos × ROI × baseline	16	2.30	0.002
**700–900 ms**			
Adverbial	2	0.89	0.412
SentPos	1	30.07	< 0.001
ROI	8	52.16	< 0.001
baseline	1	112.05	< 0.001
Adverbial × SentPos	2	1.80	0.166
Adverbial × ROI	16	1.17	0.281
Adverbial × baseline	2	1.85	0.157
SentPos × ROI	8	7.00	< 0.001
SentPos × baseline	1	1.08	0.299
ROI × baseline	8	1.17	0.311
Adverbial × SentPos × ROI	16	1.39	0.133
Adverbial × SentPos × baseline	2	2.39	0.091
Adverbial × ROI × baseline	16	0.66	0.839
SentPos × ROI × baseline	8	0.95	0.473
Adverbial × SentPos × ROI × baseline	16	0.93	0.539
**900–1,000 ms**			
Adverbial	2	1.30	0.272
SentPos	1	0.13	0.714
ROI	8	10.35	< 0.001
baseline	1	42.35	< 0.001
Adverbial × SentPos	2	1.35	0.260
Adverbial × ROI	16	8.70	< 0.001
Adverbial × baseline	2	0.62	0.536
SentPos × ROI	8	1.04	0.403
SentPos × baseline	1	5.58	0.018
ROI × baseline	8	2.13	0.030
Adverbial × SentPos × ROI	16	6.76	< 0.001
Adverbial × SentPos × baseline	2	1.71	0.182
Adverbial × ROI × baseline	16	0.94	0.524
SentPos × ROI × baseline	8	0.50	0.859
Adverbial × SentPos × ROI × baseline	16	1.41	0.127

Adverbial, idag, hemma or kanske; SentPos, sentence position, that is,V2 or V3; ROI, nine regions of interest (left/right/central for anterior/midline/posterior).

#### 100–300 ms

**Figure 7:**
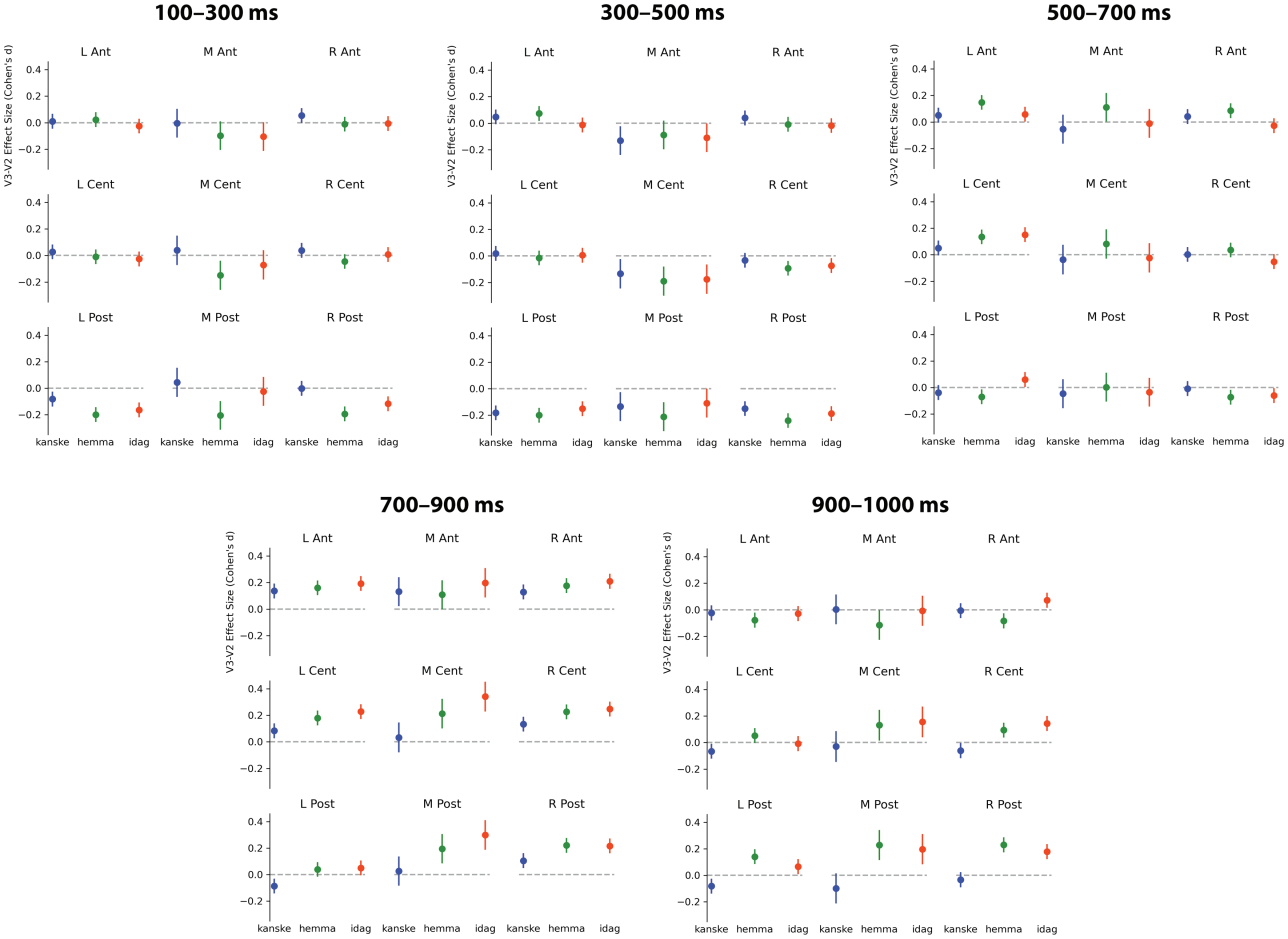
The effect sizes from the linear effects model for the effects (V2 subtracted from V3) of word order (sentence position) with each adverbial for each time window and ROI. Effect sizes (Cohen’s *d*) with 95% confidence intervals as error bars shown in blue for *kanske*, green for *hemma* and red for *idag*. L, left; M, medial; R, right; Ant, anterior; Cent, central; Post, posterior. Positive plotted up.

In the earliest time window examined, a negativity was already visible in the difference waveforms and topographic maps, particularly over left and right posterior ROIs, and extending toward the vertex of the head. The Adverbial × Sentence Position × ROI interaction was significant, *F*(16) = 1.7, *p* = 0.0388. Planned V3-V2 contrasts for each adverbial, at each ROI, are shown in Table 6 and visualised in Figure 7. Responses to critical words following all three adverbials showed a significantly greater negativity in V3 than V2 sentence positions at the left posterior ROI, at the right ROI for *hemma* and *idag* and also at the midline central and posterior ROIs for *hemma.*

**Table 6:** V3-V2 contrasts.

Adverbial	ROI	*t*	*p* (FDR BH)	Effect.size	SE.eff	Lower.CL	Upper.CL
**100–300 ms**							
*kanske*	L_Ant	0.41	0.800	0.011	0.025	−0.039	0.060
*hemma*	L_Ant	0.91	0.580	0.023	0.025	−0.027	0.073
*idag*	L_Ant	−1.00	0.534	−0.026	0.026	−0.076	0.024
*kanske*	M_Ant	−0.08	0.969	−0.004	0.052	−0.107	0.098
*hemma*	M_Ant	−1.88	0.161	−0.098	0.052	−0.201	0.004
*idag*	M_Ant	−2.00	0.137	−0.105	0.053	−0.208	−0.002
*kanske*	R_Ant	2.12	0.115	0.054	0.025	0.004	0.104
*hemma*	R_Ant	−0.45	0.800	−0.011	0.025	−0.061	0.038
*idag*	R_Ant	−0.26	0.859	−0.007	0.026	−0.057	0.043
*kanske*	L_Cent	1.06	0.534	0.027	0.025	−0.023	0.077
*hemma*	L_Cent	−0.41	0.800	−0.010	0.025	−0.060	0.039
*idag*	L_Cent	−1.03	0.534	−0.026	0.026	−0.077	0.024
*kanske*	M_Cent	0.73	0.659	0.039	0.054	−0.066	0.144
*hemma*	M_Cent	−2.82	0.019	−0.150	0.053	−0.254	−0.046
*idag*	M_Cent	−1.33	0.383	−0.071	0.053	−0.176	0.034
*kanske*	R_Cent	1.51	0.295	0.038	0.025	−0.011	0.088
*hemma*	R_Cent	−1.77	0.187	−0.045	0.025	−0.095	0.005
*idag*	R_Cent	0.29	0.859	0.007	0.026	−0.043	0.058
*kanske*	L_Post	−3.22	0.006	−0.082	0.025	−0.131	−0.032
*hemma*	L_Post	−7.82	< 0.001	−0.198	0.025	−0.248	−0.149
*idag*	L_Post	−6.35	< 0.001	−0.163	0.026	−0.214	−0.113
*kanske*	M_Post	0.84	0.598	0.044	0.053	−0.059	0.148
*hemma*	M_Post	−3.91	0.001	−0.205	0.052	−0.307	−0.102
*idag*	M_Post	−0.45	0.800	−0.024	0.053	−0.127	0.079
*kanske*	R_Post	−0.03	0.976	−0.001	0.025	−0.051	0.049
*hemma*	R_Post	−7.58	< 0.001	−0.194	0.026	−0.244	−0.143
*idag*	R_Post	−4.58	< 0.001	−0.117	0.026	−0.167	−0.067
**300–500 ms**							
*kanske*		−5.89	< 0.001	−0.073	0.012	−0.098	−0.049
*hemma*		−8.71	< 0.001	−0.108	0.012	−0.133	−0.084
*idag*		−7.41	< 0.001	−0.092	0.012	−0.117	−0.068
	L_Ant	2.40	0.021	0.035	0.015	0.006	0.064
	M_Ant	−3.65	< 0.001	−0.110	0.030	−0.169	−0.051
	R_Ant	0.27	0.830	0.004	0.015	−0.025	0.033
	L_Cent	0.22	0.830	0.003	0.015	−0.026	0.032
	M_Cent	−5.41	< 0.001	−0.167	0.031	−0.227	−0.106
	R_Cent	−4.49	< 0.001	−0.066	0.015	−0.095	−0.037
	L_Post	−12.04	< 0.001	−0.177	0.015	−0.206	−0.148
	M_Post	−4.97	< 0.001	−0.151	0.030	−0.211	−0.092
	R_Post	−13.07	< 0.001	−0.192	0.015	−0.221	−0.164
**500–700 ms**							
*kanske*	L_Ant	1.99	0.097	0.051	0.026	0.001	0.101
*hemma*	L_Ant	5.79	< 0.001	0.148	0.025	0.098	0.198
*idag*	L_Ant	2.26	0.071	0.058	0.026	0.008	0.108
*kanske*	M_Ant	−1.02	0.435	−0.054	0.053	−0.157	0.049
*hemma*	M_Ant	2.10	0.096	0.111	0.053	0.007	0.214
*idag*	M_Ant	−0.20	0.907	−0.011	0.053	−0.114	0.093
*kanske*	R_Ant	1.62	0.205	0.041	0.026	−0.009	0.091
*hemma*	R_Ant	3.35	0.006	0.085	0.025	0.035	0.135
*idag*	R_Ant	−1.08	0.423	−0.028	0.026	−0.078	0.023
*kanske*	L_Cent	1.99	0.097	0.051	0.026	0.001	0.101
*hemma*	L_Cent	5.31	< 0.001	0.135	0.025	0.085	0.185
*idag*	L_Cent	5.87	< 0.001	0.151	0.026	0.101	0.202
*kanske*	M_Cent	−0.67	0.619	−0.036	0.054	−0.142	0.070
*hemma*	M_Cent	1.52	0.227	0.081	0.054	−0.024	0.186
*idag*	M_Cent	−0.44	0.774	−0.024	0.054	−0.129	0.081
*kanske*	R_Cent	0.10	0.954	0.003	0.025	−0.047	0.053
*hemma*	R_Cent	1.42	0.249	0.036	0.026	−0.014	0.086
*idag*	R_Cent	−2.02	0.097	−0.052	0.026	−0.102	−0.002
*kanske*	L_Post	−1.50	0.227	−0.038	0.025	−0.088	0.012
*hemma*	L_Post	−2.73	0.028	−0.069	0.025	−0.119	−0.020
*idag*	L_Post	2.33	0.066	0.060	0.026	0.010	0.111
*kanske*	M_Post	−0.87	0.518	−0.046	0.053	−0.150	0.058
*hemma*	M_Post	0.04	0.966	0.002	0.053	−0.101	0.106
*idag*	M_Post	−0.67	0.619	−0.035	0.053	−0.138	0.068
*kanske*	R_Post	−0.32	0.841	−0.008	0.026	−0.058	0.042
*hemma*	R_Post	−2.85	0.024	−0.073	0.026	−0.123	−0.023
*idag*	R_Post	−2.37	0.066	−0.061	0.026	−0.111	−0.010
**700–900 ms**							
	L_Ant	11.02	< 0.001	0.163	0.015	0.134	0.192
	M_Ant	4.74	< 0.001	0.146	0.031	0.085	0.206
	R_Ant	11.59	< 0.001	0.171	0.015	0.142	0.200
	L_Cent	11.04	< 0.001	0.163	0.015	0.134	0.192
	M_Cent	6.21	< 0.001	0.196	0.031	0.134	0.257
	R_Cent	13.66	< 0.001	0.202	0.015	0.173	0.231
	L_Post	0.07	0.941	0.001	0.015	−0.028	0.030
	M_Post	5.62	< 0.001	0.174	0.031	0.113	0.234
	R_Post	12.19	< 0.001	0.181	0.015	0.152	0.210
**900–1,000 ms**							
*kanske*	L_Ant	−0.92	0.439	−0.024	0.026	−0.074	0.027
*hemma*	L_Ant	−3.05	0.006	−0.079	0.026	−0.129	−0.028
*idag*	L_Ant	−1.09	0.357	−0.028	0.026	−0.079	0.023
*kanske*	M_Ant	0.05	0.962	0.003	0.054	−0.104	0.109
*hemma*	M_Ant	−2.10	0.057	−0.115	0.055	−0.222	−0.007
*idag*	M_Ant	−0.13	0.927	−0.007	0.055	−0.115	0.100
*kanske*	R_Ant	−0.25	0.867	−0.006	0.026	−0.057	0.044
*hemma*	R_Ant	−3.24	0.004	−0.084	0.026	−0.134	−0.033
*idag*	R_Ant	2.77	0.013	0.072	0.026	0.021	0.123
*kanske*	L_Cent	−2.57	0.021	−0.066	0.026	−0.116	−0.016
*hemma*	L_Cent	2.05	0.061	0.053	0.026	0.002	0.103
*idag*	L_Cent	−0.29	0.867	−0.008	0.026	−0.059	0.044
*kanske*	M_Cent	−0.54	0.694	−0.030	0.056	−0.140	0.080
*hemma*	M_Cent	2.33	0.033	0.131	0.056	0.021	0.240
*idag*	M_Cent	2.77	0.013	0.156	0.056	0.045	0.266
*kanske*	R_Cent	−2.37	0.032	−0.061	0.026	−0.112	−0.011
*hemma*	R_Cent	3.62	0.001	0.094	0.026	0.043	0.144
*idag*	R_Cent	5.53	< 0.001	0.144	0.026	0.093	0.195
*kanske*	L_Post	−3.19	0.004	−0.082	0.026	−0.132	−0.031
*hemma*	L_Post	5.46	< 0.001	0.141	0.026	0.090	0.191
*idag*	L_Post	2.51	0.023	0.066	0.026	0.014	0.117
*kanske*	M_Post	−1.80	0.103	−0.099	0.055	−0.207	0.009
*hemma*	M_Post	4.15	< 0.001	0.227	0.055	0.120	0.335
*idag*	M_Post	3.57	0.001	0.197	0.055	0.089	0.305
*kanske*	R_Post	−1.28	0.273	−0.033	0.026	−0.084	0.018
*hemma*	R_Post	8.85	< 0.001	0.230	0.026	0.179	0.281
*idag*	R_Post	6.86	< 0.001	0.179	0.026	0.128	0.230

The first two columns in regard to the difference between V3 and V2 in the particular time window. ROI, regions of interest; L, left; R, right; M, midline; Ant, anterior; Cent, central; Post, posterior.

#### 300–500 ms

In this time window, the difference waveforms and topographic maps suggested an N400-like negativity largest over the vertex and lateral posterior sites. The Adverbial × Sentence Position × ROI interaction was not significant, however the Adverbial × Sentence Position, *F*(2) = 3.35, *p* < 0.0201 and Sentence Position × ROI, *F*(8) = 8.25, *p* < 0.0001, interactions were both significant. The Adverbial × Sentence Position interaction was attributable to a larger negativity for *hemma* than either *kanske* or *idag*, although the V3-V2 difference was significant for all three adverbials, collapsed across ROIs. The interaction of Sentence Position × ROI was due to significantly greater negativity for V3 than V2 sentences at the vertex (midline central) and all three posterior ROIs. This was true for all three adverbials, reflecting the lack of a 3-way interaction. The contrasts for both of these 2-way interactions are presented in Table 6. For consistency with other time windows, Figure 7 plots the effects from the Adverbial × Sentence Position × ROI, but caution should be used not to infer differences between ROIs as a function of adverbial.

#### 500–700 ms

In this time window, the negativity present in earlier periods appeared to be giving way to a positivity, especially at left central electrodes. The Adverbial × Sentence Position × ROI interaction was significant, *F*(16) = 3.01, *p* < 0.0001. Planned V3-V2 contrasts for each adverbial, at each ROI, are shown in Table 6 and visualised in Figure 7. The greater positivity for V3 than V2 sentence positions apparent in the ERP plots was significant for *hemma* at the left central and right anterior ROIs, while at left and right posterior ROIs the difference was significant but reversed (i.e. more negative for V3). The greater positivity for V3 was also significant for *idag* at the left central ROI.

#### 700–900 ms

This time window captured the peak of the greater positivity for V3 than V2 sentences that was apparent in the difference waveforms and topographic plots. The Adverbial × Sentence Position × ROI interaction was not significant, however, the Sentence Position × ROI interaction was significant, *F*(8) = 7.00, *p* < 0.0001. This was consistent with the observation that the positivity was present, with similar scalp distributions, for all three adverbials (although subjectively it appeared larger for *hemma* and *idag* than for *kanske*). The V3-V2 positivity was significant at all ROIs except left posterior sites.

Although there are two positive peaks visually in Figure 2 in this and the previous time window, the statistical analyses and the difference wave forms (Figures 3–6) show that the positive effect of word order 500–700 ms overlaps in timing with a negativity, while there is only a positivity in the time window 700–900 ms. Our interpretation is therefore that it is one positive effect to the word order violation (V3) and that the different distribution is due to the combination with the negativity.

#### 900–1,000 ms

In this time window, the difference waveforms and topographic maps indicated a continued positivity for V3 relative to V2 sentences, with a similar topography as in the preceding time window. The Adverbial × Sentence Position × ROI interaction was significant, *F*(16) = 6.76, *p* < 0.0001. Planned V3-V2 contrasts for each adverbial, at each ROI, are shown in Table 6 and visualised in Figure 7. The adverbials *hemma* and *idag* showed similar patterns of significance, with greater positivity for V3 than V2 at midline central and right central and all three posterior ROIs. *Hemma* also showed a significantly greater negativity for V3 than V2, at left and right anterior ROIs. In contrast to the effects for those adverbials, *kanske* was associated with more negative responses for V3 than V2, at the left central and posterior, and right central, ROIs.

### Interactions With Word Order Preference

We conducted additional linear mixed effects models, following the same procedures as above but with the inclusion of an additional behavioural variable derived from the SCT. Specifically, we included the proportion of V2 sentences produced by each participant (such that a lower proportion would indicate greater tendency to produce V3 constructions). SCT performance was included as an additional fixed effect and allowed to interact with all other fixed effects except baseline, up to the full 4-way interaction of Adverbial × Sentence Position × ROI × SCT. We did not include interactions of baseline with SCT, since SCT was a subject-level variable and did not vary within an individual as a function of experimental conditions, so there was no justification to consider its interaction with baseline. Baseline was nonetheless still included in the model, with up to the 4-way interaction Adverbial × Sentence Position × ROI × Baseline as in the analyses reported above. Here we report only the significant interactions involving SCT and one or both of Adverbial and Sentence Position, as well as ROI if present.

In the 100–300 ms time window, SCT did not interact with other variables of interest.

**Figure 8:**
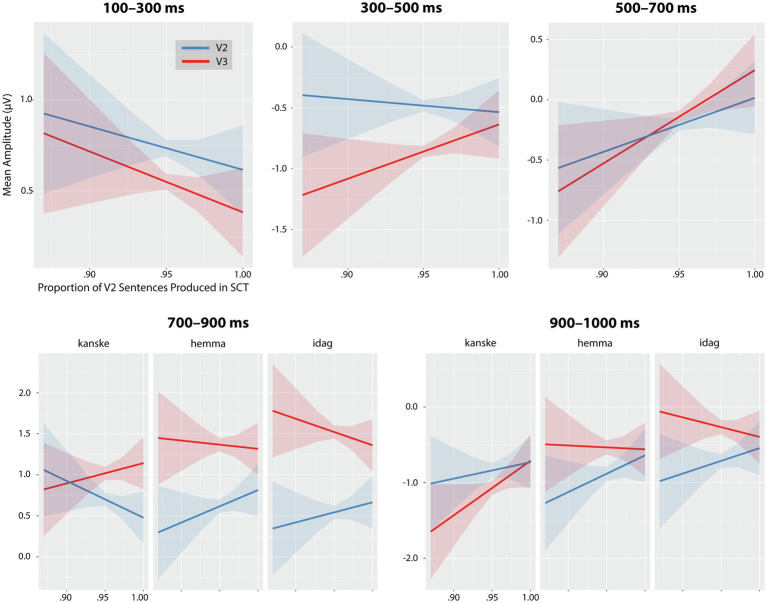
Significant interactions between proportion of produced V2 sentences in SCT and mean amplitudes for V2 (in blue) and V3 (in red) for each time window. Confidence intervals (95%) are shaded. In the first three time windows (100–300, 300–500 and 500–700 ms) the interactions are collapsed across adverbials (since adverb did not interact with sentence position or SCT) while in the two later time windows (700–900 and 900–1,000 ms) the interaction with Adverbial was significant, and so, effects are separated by adverbial.

In the 300–500 ms time window, SCT interacted with Sentence Position, *F*(1) = 6.76, *p* = 0.009. Across all adverbials and ROIs, SCT significantly modulated the size of the V3-V2 ERP difference, as shown in Figure 8. Specifically, the negativity elicited by V3 sentences relative to V2 was largest for those individuals who also produced a greater number of V3 sentences.

In the 500–700 ms time window, the SCT × Sentence Position interaction was again significant, *F*(1) = 4.48, *p* = 0.034. The relationship between SCT and ERP amplitude in this case was more subtle than in the preceding time window, however, shown in Figure 8—there was a ‘crossover’ effect such that ERP amplitudes were more negative for V3 than V2 in individuals who produced more V3 sentences, but for individuals who produced fewer V3 sentences, the pattern was reversed, with a more positive amplitude for V3 than V2.

In the 700–900 ms time window, there was a significant 3-way interaction of SCT × Adverbial × Sentence Position, *F*(2) = 5.86, *p* = 0.003. As shown in Figure 8, the greater positivity for V3 than V2 sentences was largest among participants who produced more V3 sentences, but this was true only for *hemma* (*t =* −3.45, *p* = 0.008) and *idag* (*t* = −3.94, *p* = 0.001). For *kanske*, SCT also significantly modulated the V3-V2 difference (*t* = 4.85, *p* < 0.001), but for this adverbial the positivity was greater among those who produced more V2 sentences—the opposite of the pattern observed for the other adverbials.

Finally, in the 900–1,000 ms time window, there was again a significant SCT × Adverbial × Sentence Position. The pattern of results varied by adverbial as shown in Figure 8 and was similar to the pattern observed in the preceding 700–900 ms window (greater positivity for V3 than V2 for those producing more V3 sentences) for *hemma* (*t* = −3.18, *p* = 0.018) and *idag* (*t* = −3.55, *p* = 0.005). For *kanske* (*t* = −3.02, *p* = 0.031), the pattern was different from the preceding window; in this later window, those who produced more V3 sentences showed a greater positivity for V2 than V3 sentences, while little difference was seen between V2 and V3 sentences among those producing the most V2 sentences.

To summarise the relationships observed between V3 sentence production preference and ERP effects to word orders, we can make the following generalisations. Participants who produced predominantly or exclusively V2 constructions showed little or no difference in the ERP effects for V3 and V2 sentences in the N400 time window. They did, however, show a stronger positive effect of word order with an onset of the positivity between 500 and 700 ms. Participants who produced more V3 sentences tended to show greater negativities for V3 relative to V2 sentences in the N400 time window and greater positivities for V3 in the late positivity time windows for *hemma* and *idag*. Responses to *kanske* sentences in the late positivity time windows were more complex, with greater positivities for V3 constructions in the 700–900 ms time window among those who produced predominantly or exclusively V2 constructions; but greater positivity for *V2* sentences in the 900–1,000 ms time window, for those who produced more V3 sentences.

## Discussion

This study aimed to investigate how variations in basic word order are handled behaviourally and neurocognitively by testing how native Swedish speakers process variations in basic word order (V2/V3) depending on sentence-initial adverbials. As predicted, the results revealed overall effects of V2-V3 word order in all tests. V2 was at ceiling and more frequent than V3 in the sentence completion task, confirming V2 to be an overall strong pattern in native Swedish. Similarly, V2 word order was more acceptable than V3 word order in the acceptability judgements. Neurocognitively, we found the expected biphasic N400/P600 response to V3 word orders. The presence of these effects despite the early appearance of the violation in the sentence, which gave the parser little time to build an expectation, can be an indication of the strong preference for V2 word order in Swedish.

Turning to the effects of sentence-initial adverbials we found effects in all tests, also as predicted. In the sentence completion task, V3 word order was more frequent after initial *kanske* ‘maybe’ than with the temporal and spatial adverbials. Similarly, V3 word order was most acceptable with *kanske*. In addition, participants were more likely to accept V3 sentences with *hemma* than with *idag,* possibly due to the unusual occurrence of *hemma* in sentence-initial position, as revealed by the corpus study (cf. Jörgensen, 1976). The ERP analyses with the three adverbials showed a negativity 100–500 ms followed by positivities 500–1,000 ms that differed in amplitudes and onsets. Overall, these results replicated the previously reported biphasic response to V3 following long prefields (Andersson et al., 2019). More interestingly, however, the effects were stronger for *hemma* and *idag* and weaker or absent for *kanske*. Specifically, a negativity with an N400 distribution was strongest for *hemma* and *idag* in the early time window, when only a left posterior negativity was elicited with V3 and *kanske*. The N400 was followed by a positivity that was initially strongest over anterior and central sites (see Figure 6; Table 6), as previously reported in relation to syntactic integration and complex sentences (e.g. Friederici et al., 2002), followed by a typical centro-parietal P600 distribution, reflecting sentence reanalysis (e.g. Kaan et al., 2000; Vos et al., 2001; Fiebach et al., 2002; Kaan and Swaab, 2003). Importantly, the first positivity onset was in an earlier time window for *idag* and *hemma* (500–700 ms) compared to *kanske* (700–900 ms), while the P600 (positivity was strongest over centro-parietal sites) was not present for *kanske.* These results suggest a different processing of V3 with *kanske* compared to *hemma* and *idag*.

The correlation analyses examining the relationship between the neurocognitive effects and the production of correct V2 word order can shed light on the underlying nature of the effects (see Figure 8). Recall that during the first three time windows (100–700 ms), the SCT was related to the effects of word order (V2/V3), while in the two subsequent time windows the SCT was related to the effects to word order for each adverbial separately. In sum, the results show a greater likelihood of (1) a biphasic response (N400/P600) in participants who produce more V3 word orders, but only a P600 in those who produce predominantly or exclusively V2 word orders; (2) a longer latency of the P600 with the adverbs *hemma* and *idag* with more V3 production; and as well (3) a late P600 for V2 with the adverb *kanske* for those producing more V3.

Overall, individuals who produced more V3 word orders showed an increased N400 (300–700 ms), suggesting a greater reliance on lexical or semantic processes (Osterhout, 1997; Kimppa et al., 2019) in response to a violation of word expectancy, rather than a syntactic reanalysis of the sentence (Dröge et al., 2016; Kuperberg et al., 2020). This negativity was not present in individuals who did not produce any V3 word order. In fact, participants who produced more V2 word orders showed a stronger positivity already between 500 and 700 ms, an effect that remained in the following time window (700–900 ms) only in sentences with *kanske*. This later positivity with an anterior distribution occurring with *kanske* might indicate uncertainty and increased working memory load (see Kuperberg et al., 2020 for a discussion on late anterior positivities). The same pattern was present in individuals who produced more V3 word orders, but with longer latency (first between 700 and 900 ms) and the positivities were restricted to *hemma* and *idag*. This suggests that there was a reanalysis of the V3 word orders with these two adverbials also with higher production of V3 but with longer latency than for participants with lower production of V3. In addition, correlational analyses indicated a positivity with *kanske* also in the subsequent time window with the production of more V3 sentences but for this adverbial the P600 effect that suggest a reanalysis was related to the presentation of correctly formed V2 sentences. Thus, the results suggest that participants who produced more V3 processed V2 sentences with *kanske* as violations of the anticipated word order which therefore needed to be reanalysed.

Thus, the results reveal individual differences in the timing and amplitude of the effects (N400 or P600 effects), replicating previous studies that have found a biphasic response in group averages which in subsequent analyses are revealed to reflect individual differences (Choudhary et al., 2009; McLaughlin et al., 2010; Tanner et al., 2014; Tanner and Hell, 2014; Kim et al., 2018). However, in addition to individual differences in response to V3 in general, results also showed differences in response to V3 by adverbial. In sum, the behavioural results and the ERP results for the group aligned with the predictions, while the correlation analyses of behavioural (production) results and ERP effects revealed some unexpected findings. Although we expected a P600 effect of word order to be attenuated for *kanske*, indicating more uncertainty since both V2 and V3 occur naturally, this indicator of sentence reanalysis varied with the amount of V3 sentences participants produced. While those who predominantly or exclusively produced V2 showed greater positivities to V3 *kanske* sentences with longer latencies than with the other two adverbials, those who produced more V3 sentences showed a similar positivity for *kanske* with even later onset and crucially to V2 *kanske* sentences rather than V3 sentences. A tentative and speculative explanation may be that naturally occurring word order variation in a language leads to prolonged processing. When speakers additionally *produce* variant structures, uncertainty and effort in processing affect the timing of the reanalysis with some adverbials even of sentences normatively regarded as ‘correct’.

In line with the acceptability judgement results, there were some differences also between the ERP effects for *idag* and *hemma*. The negativity was stronger and more distributed temporally and spatially for *hemma* than *idag* (Figure 6; Table 6). Also, the positivity was more spatially distributed with *hemma*. As previous studies report on temporally and spatially focal effects as being related to higher proficiency and ease of processing (e.g. Pakulak and Neville, 2010), the temporally and spatially distributed effect could be a reflection of the very few occurrences of sentence-initial *hemma* in the corpus study.

In conclusion, the combined behavioural and neurocognitive results indicate that V2-V3 variations in Swedish are more behaviourally acceptable with some adverbials than with others (here *kanske* ‘maybe’), but also that such sentences lead to delayed processing that require more effort in processing in comparison to sentences starting with other adverbials (here *hemma* and *idag*). These results are commensurate with other studies showing that structures, which are traditionally labelled as violations but that occur naturally in production, are dealt with differently from those traditionally labelled as ‘correct’ (cf. Duffield et al., 2002, 2007; Hubers et al., 2016, 2020). Importantly, the processing of word order varies depending on individual word order production. Thus, native basic word order processing is not uniform.

## Data Availability Statement

The datasets presented in this study can be found in online repositories. The names of the repository/repositories and accession number(s) can be found at: Open Science Foundation, https://dx.doi.org/10.17605/OSF.IO/5VN2Y.

## Ethics Statement

The studies involving human participants were reviewed and approved by Ethics Review Board of Southern Sweden (approval number 2012/9). The participants provided their written informed consent to participate in this study.

## Author Contributions

SS, MG, and AA conceived and designed the study. SS and AA performed the experiments and collected the data. Data analyses were divided by the authors. SS conducted the behavioural analyses. AA and AN conducted the ERP analyses. MG conducted the corpus data analyses. All authors contributed to the interpretation of the results and the writing of the manuscript and approved the final version of the manuscript for submission.

## Funding

We gratefully acknowledge funding from the Swedish Research Council, grant number 21-2010-2114 to MG (Swedish Word Order Processing in Second Language Learners and Native Speakers: A Psycholinguistic and Neurocognitive Approach). AA is supported by Riksbankens jubileumsfond P17-0535:1. AN is supported by a Discovery Grant from the Natural Sciences and Engineering Research Council of Canada (NSERC). RGPIN-2017-05340.

## Conflict of Interest

The authors declare that the research was conducted in the absence of any commercial or financial relationships that could be construed as a potential conflict of interest.

## Publisher’s Note

All claims expressed in this article are solely those of the authors and do not necessarily represent those of their affiliated organizations, or those of the publisher, the editors and the reviewers. Any product that may be evaluated in this article, or claim that may be made by its manufacturer, is not guaranteed or endorsed by the publisher.

## Footnotes

^1^The […] in both examples (1a) and (1b) are place holders for the Swedish negation particle *inte ‘*not*’*. In the Swedish Academy Grammar (SAG) examples are often presented in negated form in order to fully illustrate possible word order phenomena. As negation is not discussed in this paper, the sentence is presented in the affirmative mode.

^2^ https://dx.doi.org/10.17605/OSF.IO/5VN2Y

^3^Although other R packages are often used for linear mixed effects analyses, such as *lme4*, we used *mgcv*’s *bam* because it is parallelized and runs considerably faster, and does not suffer convergence errors. By setting *bam*’s smooth terms to “re” we are able to include random effects.

^4^The model that we fit that achieved the lowest AIC actually had a more complex random effects structure, comprising random slopes for channel by subject, and random slopes of sentence (i.e., item) by subject. However, the computational complexity of this model made it infeasible to derive all of the desired simple effects contrasts needed to interpret and visualize the model. For this reason, we instead used a model with the same fixed effects structure, but a simpler random effects structure comprising only random intercepts for subjects and random intercepts for sentences. The results of these two models, in terms of which interactions achieved statistical significance, were very similar, and in our experience, more complex random effects structures lead to greater explained variance of the models, but negligible differences in the pattern or statistical significance of the results.

## References

[ref1] Akaike H. (1973). “Information theory and an extension of the maximum likelihood principle.” in 2nd International Symposium on Information Theory, Tsahkadsor, Armenia, USSR, September 2–8, 1971. eds. Petrov B. N. Csáki F. (Budapest: Akadémiai Kiadó), 267–281. Republished in S. Kotz and N. L. Johnson (eds.) (1992), *Breakthroughs in Statistics, I,* Springer-Verlag, 610–624.

[ref2] Alday P. M. (2019). How much baseline correction do we need in ERP research? Extended GLM model can replace baseline correction while lifting its limits. Psychophysiology 56:e13451. doi: 10.1111/psyp.13451, PMID: 31403187

[ref3] Allen D. (1992). Oxford Placement Test. Oxford: Oxford University Press.

[ref4] Almor A. de Carvalho Maia J. Cunha Lima M. L. Vernice M. Gelormini-Lezama C. (2017). Language processing, acceptability, and statistical distribution: a study of null and overt subjects in Brazilian Portuguese. J. Mem. Lang. 92, 98–113. doi: 10.1016/j.jml.2016.06.001

[ref5] Andersson A. Sayehli S. Gullberg M. (2019). Language background affects online word order processing in a second language but not offline. Biling.: Lang. Cogn. 22, 802–825. doi: 10.1017/S1366728918000573

[ref6] Andréasson M. (2007). Satsadverbial, ledföljd och informationsdynamik i svenskan. Doctoral thesis. University of Gothenburg, Gothenburg.

[ref7] Benjamini Y. Hochberg Y. (1995). Controlling the false discovery rate: a practical and powerful approach to multiple testing. J. R. Stat. Soc. Ser. B Methodol. 57, 289–300. doi: 10.1111/j.2517-6161.1995.tb02031.x

[ref8] Bohnacker U. (2006). When Swedes begin to learn German: from V2 to V2. Second. Lang. Res. 22, 443–486. doi: 10.1191/0267658306sr275oa

[ref9] Bolander M. (1988). “Nu ja hoppas inte så mycke: om inversion och placering av negation och adverb i svenska som andraspråk,” in Första symposiet om svenska som andraspråk: Föredrag om språk, språkinlärning och interaktion. Vol. 1. eds. Hyltenstam K. Lindberg I. (Stockholm: Stockholm University, Centre for Research on Bilingualism), 203–214.

[ref10] Borin L. Forsberg M. Roxendal J. (2012). “Korp – the corpus infrastructure of Språkbanken.” in Proceedings of LREC 2012; May 21–27, 2012; Istanbul: ELRA, 474–478.

[ref11] Brouwer H. Crocker M. W. (2017). On the proper treatment of the N400 and P600 in language comprehension. Front. Psychol. 8:1327. doi: 10.3389/fpsyg.2017.01327, PMID: 28824506 PMC5539129

[ref12] Brouwer H. Crocker M. W. Venhuizen N. J. Hoeks J. C. J. (2017). A neurocomputational model of the N400 and the P600 in language processing. Cogn. Sci. 41, 1318–1352. doi: 10.1111/cogs.12461 28000963 PMC5484319

[ref13] Brouwer H. Delogu F. Venhuizen N. J. Crocker M. W. (2021). Neurobehavioral correlates of surprisal in language comprehension: a neurocomputational model. Front. Psychol. 12:615538. doi: 10.3389/fpsyg.2021.615538, PMID: 33643143 PMC7905034

[ref14] Brouwer H. Fitz H. Hoeks J. (2012). Getting real about semantic illusions: rethinking the functional role of the P600 in language comprehension. Brain Res. 1446, 127–143. doi: 10.1016/j.brainres.2012.01.055, PMID: 22361114

[ref15] Carrasco-Ortíz H. Velázquez Herrera A. Jackson-Maldonado D. Avecilla Ramírez G. N. Silva Pereyra J. Wicha N. Y. Y. (2017). The role of language similarity in processing second language morphosyntax: evidence from ERPs. Int. J. Psychophysiol. 117, 91–110. doi: 10.1016/j.ijpsycho.2017.04.008, PMID: 28456582

[ref16] Choudhary K. K. Schlesewsky M. Roehm D. Bornkessel-Schlesewsky I. (2009). The N400 as a correlate of interpretively relevant linguistic rules: evidence from Hindi. Neuropsychologia 47, 3012–3022. doi: 10.1016/j.neuropsychologia.2009.05.009, PMID: 19465035

[ref19] den Ouden D. B. Bastiaanse R. (2009). The electrophysiological manifestation of Dutch verb second violations. J. Psycholinguist. Res. 38, 201–219. doi: 10.1007/s10936-009-9106-6, PMID: 19330528

[ref20] Dröge A. Fleischer J. Schlesewsky M. Bornkessel-Schlesewsky I. (2016). Neural mechanisms of sentence comprehension based on predictive processes and decision certainty: electrophysiological evidence from non-canonical linearizations in a flexible word order language. Brain Res. 1633, 149–166. doi: 10.1016/j.brainres.2015.12.045, PMID: 26740402

[ref21] Duffield N. Matsuo A. Roberts L. (2007). Acceptable ungrammaticality in sentence matching. Second. Lang. Res. 23, 155–178. doi: 10.1177/0267658307076544

[ref22] Duffield N. White L. Bruhn de Garavito J. Montrul S. Prévost P. (2002). Clitic placement in L2 French: evidence from sentence matching. J. Linguist. 38, 1–37. doi: 10.1017/S0022226702001688

[ref23] Erdocia K. Laka I. Mestres-Missé A. Rodriguez-Fornells A. (2009). Syntactic complexity and ambiguity resolution in a free word order language: behavioral and electrophysiological evidences from Basque. Brain Lang. 109, 1–17. doi: 10.1016/j.bandl.2008.12.003, PMID: 19223065

[ref24] Fanselow G. Frisch S. (2006). “Effects of processing difficulty on judgements of acceptability,” in Gradience in Grammar: Generative Perspectives. eds. Fanselow G. Vogel R. Schlesewsky M. (Oxford: Oxford University Press), 291–316.

[ref25] Fiebach C. J. Schlesewsky M. Friederici A. D. (2002). Separating syntactic memory costs and syntactic integration costs during parsing: the processing of German WH-questions. J. Mem. Lang. 47, 250–272. doi: 10.1016/S0749-596X(02)00004-9

[ref26] Frazier L. (1987). “Sentence processing: a tutorial review,” in Attention and performance: *The psychology of reading*. Vol. 12. ed. Coltheart M. (Hillsdale, NJ: Lawrence Erlbaum Associates), 559–586.

[ref27] Freywald U. Cornips L. Ganuza N. Nistov I. Opsahl T. (2015). “Beyond verb second – a matter of novel informationstructural effects? Evidence from Norwegian, Swedish, German and Dutch,” in Language, Youth and Identity in the 21st Century: Linguistic Practices across Urban Spaces. eds. Nortier J. Bente A. S. (Cambridge: Cambridge University Press), 73–92.

[ref28] Friederici A. D. (2002). Towards a neural basis of auditory sentence processing. Trends Cogn. Sci. 6, 78–84. doi: 10.1016/S1364-6613(00)01839-8, PMID: 15866191

[ref29] Friederici A. D. Hahne A. Saddy D. (2002). Distinct neurophysiological patterns reflecting aspects of syntactic complexity and syntactic repair. J. Psycholinguist. Res. 31, 45–63. doi: 10.1023/A:1014376204525, PMID: 11924839

[ref30] Friederici A. D. Pfeifer E. Hahne A. (1993). Event-related brain potentials during natural speech processing: effects of semantic, morphological and syntactic violations. Cogn. Brain Res. 1, 183–192. doi: 10.1016/0926-6410(93)90026-2, PMID: 8257874

[ref31] Friederici A. D. Steinhauer K. Mecklinger A. Meyer M. (1998). Working memory constraints on syntactic ambiguity resolution as revealed by electrical brain responses. Biol. Psychol. 47, 193–221. doi: 10.1016/S0301-0511(97)00033-1, PMID: 9564450

[ref001] Fromont L. A. Steinhauer K. Royle P. (2020). Verbing nouns and nouning verbs: Using a balanced design provides ERP evidence against “syntax-first” approaches to sentence processing. PLoS One 15:e0229169. doi: 10.1111/j.1749-818X.2007.00037.x, PMID: 32168357 PMC7069651

[ref32] Ganuza N. (2008). Syntactic variation in the Swedish of adolescents in multilingual urban settings: subject-verb order in declaratives, questions and subordinate clauses. Doctoral thesis. Stockholm University, Stockholm.

[ref33] Gramfort A. Luessi M. Larson E. Engemann D. A. Strohmeier D. Brodbeck C.. (2013). MEG and EEG data analysis with MNE-python. Front. Neurosci. 7:267. doi: 10.3389/fnins.2013.00267, PMID: 24431986 PMC3872725

[ref34] Gullberg M. Indefrey P. (2003). Language Background Questionnaire. The Dynamics of Multilingual Processing. Nijmegen: Max Planck Institute for Psycholinguistics.

[ref35] Hagoort P. Brown C. Groothusen J. (1993). The syntactic positive shift (SPS) as an ERP measure of syntactic processing. Lang. Cogn. Process. 8, 439–483. doi: 10.1080/01690969308407585

[ref36] Hagoort P. Indefrey P. (2014). The neurobiology of language beyond single words. Annu. Rev. Neurosci. 37, 347–362. doi: 10.1146/annurev-neuro-071013-013847, PMID: 24905595

[ref38] Hahne A. Friederici A. D. (2002). Differential task effects on semantic and syntactic processes as revealed by ERPs. Cogn. Brain Res. 13, 339–356. doi: 10.1016/S0926-6410(01)00127-6, PMID: 11918999

[ref39] Håkansson G. (1988). ““Hungry I am - breakfast I want”. On the acquisition of inverted word order in Swedish,” in Working Papers. Vol. 33. eds. Horne M. Svantesson J-O. (Lund: Lund University, Department of General Linguistics), 123–130.

[ref40] Håkansson G. Nettelbladt U. (1996). “Similarities between SLI and L2 children. Evidence from the acquisition of Swedish word order,” in Children's Language. Vol. 9. eds. Gilbert J. Johnson C. (Mahwah: New Jersey: Lawrence Erlbaum & Associates), 135–151.

[ref41] Häussler J. Grant M. Fanselow G. Frazier L. (2015). Superiority in English and German: cross-language grammatical differences? Syntax 18, 235–265. doi: 10.1111/synt.12030

[ref42] Holcomb P. J. Neville H. J. (1991). Natural speech processing: an analysis using event-related brain potentials. Psychobiology 19, 286–300. doi: 10.3758/BF03332082, PMID: 34149375

[ref43] Hollingshead A. (1975). Four Factor Index of Social Status. New Haven: Yale University Department of Sociology.

[ref44] Hopp H. (2006). Syntactic features and reanalysis in near-native processing. Second. Lang. Res. 22, 369–397. doi: 10.1191/0267658306sr272oa

[ref45] Hörberg T. Koptjevskaja-Tamm M. Kallioinen P. (2013). The neurophysiological correlate to grammatical function reanalysis in Swedish. Lang. Cogn. Process. 28, 388–416. doi: 10.1080/01690965.2011.651345

[ref46] Hubers F. Redl T. de Vos H. Reinarz L. de Hoop H. (2020). Processing prescriptively incorrect comparative particles: evidence from sentence-matching and eye-tracking. Front. Psychol. 11:186. doi: 10.3389/fpsyg.2020.00186, PMID: 32116969 PMC7034421

[ref47] Hubers F. Snijders T. M. de Hoop H. (2016). How the brain processes violations of the grammatical norm: an fMRI study. Brain Lang. 163, 22–31. doi: 10.1016/j.bandl.2016.08.006, PMID: 27639117

[ref48] Hyltenstam K. (1977). Implicational patterns in interlanguage syntax variation. Lang. Learn. 27, 383–411. doi: 10.1111/j.1467-1770.1977.tb00129.x

[ref49] Hyltenstam K. (1978). “Variability in interlanguage system,” in Working papers. Vol. 18 Oct 7, 2000; Lund: Department of General Linguistics, Lund University, 1–79.

[ref50] Hyönä J. Hujanen H. (1997). Effects of case marking and word order on sentence parsing in Finnish: an eye fixation analysis. Q. J. Exp. Psychol. 50, 841–858. doi: 10.1080/713755738

[ref51] Hyvärinen A. (1999). Fast and robust fixed-point algorithms for independent component analysis. IEEE Trans. Neural Netw. 10, 626–634. doi: 10.1109/72.761722, PMID: 18252563

[ref52] Isel F. Hahne A. Maess B. Friederici A. D. (2007). Neurodynamics of sentence interpretation: ERP evidence from French. Biol. Psychol. 74, 337–346. doi: 10.1016/j.biopsycho.2006.09.003, PMID: 17011692

[ref53] Jörgensen N. (1976). Meningsbyggnaden i talad svenska. Lund: Studentlitteratur AB.

[ref54] Josefsson G. (2003). “Input and output: sentence patterns in child and adult grammar,” in The Acquisition of Swedish Grammar. eds. Josefsson G. Platzack C. Håkansson G. (Amsterdam: Benjamins), 95–133.

[ref55] Kaan E. (1997). Processing subject-object ambiguities in Dutch. Doctoral thesis. Rijksuniversiteit Groningen

[ref002] Kaan E. (2007). Event-related potentials and language processing: a brief overview. Lang. Linguist. Compass 1, 571–591. doi: doi-org.ezp.sub.su.se/10.1111/j.1749-818X.2007.00037.x

[ref56] Kaan E. Harris A. Gibson E. Holcomb P. (2000). The P600 as an index of syntactic integration difficulty. Lang. Cogn. Process. 15, 159–201. doi: 10.1080/016909600386084, PMID: 15722211

[ref57] Kaan E. Swaab T. Y. (2003). Repair, revision, and complexity in syntactic analysis: an electrophysiological differentiation. J. Cogn. Neurosci. 15, 98–110. doi: 10.1162/089892903321107855, PMID: 12590846

[ref58] Kim A. E. Oines L. Miyake A. (2018). Individual differences in verbal working memory underlie a tradeoff between semantic and structural processing difficulty during language comprehension: an ERP investigation. J. Exp. Psychol. Learn. Mem. Cogn. 44, 406–420. doi: 10.1037/xlm0000457, PMID: 28933902

[ref59] Kimppa L. Shtyrov Y. Hut S. C. A. Hedlund L. Leminen M. Leminen A. (2019). Acquisition of L2 morphology by adult language learners. Cortex 116, 74–90. doi: 10.1016/j.cortex.2019.01.012, PMID: 30832994

[ref60] Kotsinas U.-B. (1998). “Language contact in Rinkeby, an immigrant suburb,” in Jugendsprache – Langue Des Jeunes – Youth Language. Linguistische Und Soziolinguistische Perspektiven. eds. Androutsopoulos J. K. Scholz A. (Frankfurt am Main: Peter Lang D), 125–148.

[ref61] Kuperberg G. R. Brothers T. Wlotko E. W. (2020). A tale of two positivities and the N400: distinct neural signatures are evoked by confirmed and violated predictions at different levels of representation. J. Cogn. Neurosci. 32, 12–35. doi: 10.1162/jocn_a_01465, PMID: 31479347 PMC7299186

[ref62] Kutas M. Federmeier K. D. (2000). Electrophysiology reveals semantic memory use in language comprehension. Trends Cogn. Sci. 4, 463–470. doi: 10.1016/S1364-6613(00)01560-6, PMID: 11115760

[ref63] Kutas M. Hillyard S. A. (1980). Reading senseless sentences: brain potentials reflect semantic incongruity. Science 207, 203–205. doi: 10.1126/science.7350657, PMID: 7350657

[ref64] Kutas M. Hillyard S. A. (1984). Brain potentials during reading reflect word expectancy and semantic association. Nature 307, 161–163. doi: 10.1038/307161a0, PMID: 6690995

[ref65] Lau E. F. Phillips C. Poeppel D. (2008). A cortical network for semantics: [de]constructing the N400. Nat. Rev. Neurosci. 9, 920–933. doi: 10.1038/nrn2532, PMID: 19020511

[ref68] MacDonald M. C. Hsiao Y. (2018). “Sentence comprehension,” in The Oxford Handbook of Psycholinguistics. 2nd Edn. eds. Rueschemeyer S.-A. Gaskell M. G. (Oxford: Oxford University Press), 170–196.

[ref69] MacWhinney B. Bates E. Kliegl R. (1984). Cue validity and sentence interpretation in English, German, and Italian. J. Verbal Learn. Verbal Behav. 23, 127–150. doi: 10.1016/S0022-5371(84)90093-8, PMID: 3651807

[ref70] McLaughlin J. Tanner D. Pitkänen I. Frenck-Mestre C. Inoue K. Valentine G.. (2010). Brain potentials reveal discrete stages of L2 grammatical learning. Lang. Learn. 60, 123–150. doi: 10.1111/j.1467-9922.2010.00604.x

[ref71] Meng M. Bader M. (2000). Ungrammaticality detection and garden path strength: evidence for serial parsing. Lang. Cogn. Process. 15, 615–666. doi: 10.1080/016909600750040580

[ref72] Mishra R. K. Pandey A. Srinivasan N. (2011). Revisiting the scrambling complexity hypothesis in sentence processing: a self-paced reading study on anomaly detection and scrambling in Hindi. Read. Writ. 24, 709–727. doi: 10.1007/s11145-010-9255-x

[ref73] Mueller J. L. Oberecker R. Friederici A. D. (2009). Syntactic learning by mere exposure. An ERP study in adult learners. BMC Neurosci. 10:89. doi: 10.1186/1471-2202-10-89, PMID: 19640301 PMC2726980

[ref74] Neville H. J. Nicol J. L. Barss A. Forster K. I. Garrett M. F. (1991). Syntactically based sentence processing classes: evidence from event-related brain potentials. J. Cogn. Neurosci. 3, 151–165. doi: 10.1162/jocn.1991.3.2.151, PMID: 23972090

[ref75] Oldfield R. (1971). The assessment and analysis of handedness: the Edinburgh inventory. Neuropsychologia 9, 97–113. 5146491 10.1016/0028-3932(71)90067-4

[ref76] Osterhout L. (1997). On the brain response to syntactic anomalies: manipulations of word position and word class reveal individual differences. Brain Lang. 59, 494–522. doi: 10.1006/brln.1997.1793, PMID: 9299074

[ref77] Osterhout L. Holcomb P. J. (1992). Event-related brain potentials elicited by syntactic anomaly. J. Mem. Lang. 31, 785–806. doi: 10.1016/0749-596X(92)90039-Z, PMID: 32114146

[ref78] Osterhout L. Holcomb P. J. (1993). Event-related potentials and syntactic anomaly: evidence of anomaly detection during the perception of continuous speech. Lang. Cogn. Process. 8, 413–437. doi: 10.1080/01690969308407584

[ref79] Osterhout L. Holcomb P. J. Swinney D. A. (1994). Brain potentials elicited by garden-path sentences: evidence of the application of verb information during parsing. J. Exp. Psychol. Learn. Mem. Cogn. 20, 786–803. doi: 10.1037//0278-7393.20.4.786, PMID: 8064247

[ref80] Osterhout L. McLaughlin J. Pitkänen I. Frenck-Mestre C. Molinaro N. (2006). Novice learners, longitudinal designs, and event-related potentials: a means for exploring the neurocognition of second language processing. Lang. Learn. 56, 199–230. doi: 10.1111/j.1467-9922.2006.00361.x

[ref81] Osterhout L. Nicol J. (1999). On the distinctiveness, independence, and time course of the brain responses to syntactic and semantic anomalies. Lang. Cogn. Process. 14, 283–317. doi: 10.1080/016909699386310

[ref82] Ostrosky-Solis F. Rigalt C. Perez M. Marcos J. (1996). Brain-potentials (ERPs) and syntactic comprehension: effects of thematic role order. Brain Cogn. 30, 297–300.

[ref83] Pakulak E. Neville H. J. (2010). Proficiency differences in syntactic processing in monolingual native speakers indexed by event-related brain potentials. J. Cogn. Neurosci. 22, 2728–2744. doi: 10.1162/jocn.2009.21393, PMID: 19925188 PMC2891257

[ref84] Parole corpus (2012). Språkbanken Text. Department of Swedish, Multilingualism, Language Technology. University of Gothenburg, Sweden. Available at: https://spraakbanken.gu.se/resurser/parole (Accessed October 23, 2012).

[ref86] Platzack C. (1998). Svenskans inre grammatik - det minimalistiska programmet. Lund: Studentlitteratur AB.

[ref87] Quist P. (2008). Sociolinguistic approaches to multiethnolect: language variety and stylistic practice. Int. J. Biling. 12, 43–61. doi: 10.1177/13670069080120010401

[ref89] Rayson P. (2018). Log-likelihood and effect size calculator. Available at: http://ucrel.lancs.ac.uk/llwizard.html (Accessed November 14, 2018).

[ref88] R Core Team (2021). R: A language and environment for statistical computing. R Foundation for Statistical Computing, Vienna, Austria. Available at: https://www.R-project.org/ (Accessed September 27, 2021).

[ref90] Rayson P. Garside R. (2000). “Comparing corpora using frequency profiling.” in Workshop on Comparing Corpora. Oct 7, 2000 Vol. 9. eds. Kilgarriff A. Berber Sardinha T. (Hong Kong: Association for Computational Linguistics (ACL)), 1–6.

[ref91] Rösler F. Pechmann T. Streb J. Röder B. Hennighausen E. (1998). Parsing of sentences in a language with varying word order variations of processing demands are revealed by event-related brain potentials. J. Mem. Lang. 38, 150–176. doi: 10.1006/jmla.1997.2551

[ref93] Schlesewsky M. Bornkessel I. Meyer M. (2002). Why a “word order difference” is not always a “word order” difference: a reply to Weyerts, Penke, Münte, Heinze, and Clahsen. J. Psycholinguist. Res. 31, 437–445. doi: 10.1023/A:1021209818415, PMID: 12528426

[ref94] Schneider W. Eschman A. Zuccolotto A. (2012). E-prime reference guide. Pittsburgh: Psychology Software Tools Inc.

[ref95] Schriefers H. Friederici A. D. Kuhn K. (1995). The processing of locally ambiguous relative clauses in German. J. Mem. Lang. 34, 499–520. doi: 10.1006/jmla.1995.1023, PMID: 26799065

[ref97] Steinhauer K. Drury J. E. (2012). On the early left-anterior negativity (ELAN) in syntax studies. Brain Lang. 120, 135–162. doi: 10.1016/j.bandl.2011.07.001, PMID: 21924483

[ref98] Steinhauer K. Drury J. E. Portner P. Walenski M. Ullman M. T. (2010). Syntax, concepts, and logic in the temporal dynamics of language comprehension: evidence from event-related potentials. Neuropsychologia 48, 1525–1542. doi: 10.1016/j.neuropsychologia.2010.01.013, PMID: 20138065 PMC2862874

[ref99] Swaab T. Y. Ledoux K. Camblin C. C. Boudewyn M. A. (2012). “Language-related ERP components,” in The Oxford Handbook of Event-Related Potential Components. eds. Luck S. J. Kappenman E. S. (Oxford: Oxford University Press), 397–439.

[ref100] Swedex (2012). Swedish Examinations. Available at: https://www.folkuniversitetet.se/in-english/swedex-swedish-examinations/ (Accessed March 27, 2012).

[ref101] Tanner D. Hell J. G. (2014). ERPs reveal individual differences in morphosyntactic processing. Neuropsychologia 56, 289–301. doi: 10.1016/j.neuropsychologia.2014.02.002, PMID: 24530237

[ref102] Tanner D. Inoue K. Osterhout L. (2014). Brain-based individual differences in online L2 grammatical comprehension. Biling. Lang. Cogn. 17, 277–293. doi: 10.1017/S1366728913000370

[ref103] Teleman U. Hellberg S. Andersson E. (1999). Svenska Akademiens grammatik. Vol. 4. Stockholm: Norstedts Akademiska Förlag.

[ref104] Van Petten C. Luka B. J. (2012). Prediction during language comprehension: benefits, costs, and ERP components. Int. J. Psychophysiol. 83, 176–190. doi: 10.1016/j.ijpsycho.2011.09.015, PMID: 22019481

[ref105] Venhuizen N. J. Crocker M. W. Brouwer H. (2019). Expectation-based comprehension: modeling the interaction of world knowledge and linguistic experience. Discourse Process. 56, 229–255. doi: 10.1080/0163853X.2018.1448677

[ref106] Vos S. H. Gunter T. C. Schriefers H. Friederici A. D. (2001). Syntactic parsing and working memory: the effects of syntactic complexity, reading span, and concurrent load. Lang. Cogn. Process. 16, 65–103. doi: 10.1080/01690960042000085

[ref107] Walkden G. (2017). Language contact and V3 in Germanic varieties new and old. J. Comp. Ger. Linguist. 20, 49–81. doi: 10.1007/s10828-017-9084-2

[ref108] Weber-Fox C. Neville H. J. (1996). Maturational constraints on functional specializations for language processing: ERP and behavioral evidence in bilingual speakers. J. Cogn. Neurosci. 8, 231–256. doi: 10.1162/jocn.1996.8.3.231, PMID: 23968150

[ref109] Westman M. (1974). Bruksprosa. Lund: Liber läromedel - Gleerups.

[ref110] Weyerts H. Penke M. Münte T. Heinze H.-J. Clahsen H. (2002). Word order in sentence processing: an experimental study of verb placement in German. J. Psycholinguist. Res. 31, 211–268. doi: 10.1023/A:1015588012457, PMID: 12092710

[ref111] Wickens T. D. (2002). Elementary Signal Detection Theory. Oxford: Oxford University Press.

[ref112] Wiese H. (2009). Grammatical innovation in multiethnic urban Europe: new linguistic practices among adolescents. Lingua 119, 782–806. doi: 10.1016/j.lingua.2008.11.002

[ref113] Wood S. N. (2017). Generalized Additive Models: An Introduction with R. 2nd Edn. Chapman and Hall/CRC.

[ref114] Yamada Y. Neville H. J. (2007). An ERP study of syntactic processing in English and nonsense sentences. Brain Res. 1130, 167–180. doi: 10.1016/j.brainres.2006.10.052, PMID: 17173867 PMC1868703

[ref115] Yamashita H. (1997). The effects of word-order and case marking information on the processing of Japanese. J. Psycholinguist. Res. 26, 163–188. doi: 10.1023/A:1025009615473

