# Supplementary material for: Native Word Order Processing Is Not Uniform: An ERP Study of Verb-Second Word Order
Source: Front Psychol. 2022 Mar 30;13:668276. doi: 10.3389/fpsyg.2022.668276 (PMC9006952; doi:10.3389/fpsyg.2022.668276)
Supplement: Supplementary file 1 [file Data_Sheet_1.pdf]

Supplementary Materials

1 Stimuli lists

1.1 Sentences for the sentence completion task

Straight lines indicate how sentences were chunked. Sentences are here presented in targeted word order. Note that the question mark was part of the lead-in fragment. For presentation purposes: subjects in bold, verbs underlined.

1. Kanske | målade | **pojken**
   Maybe painted boy$_{\text{DEF}}$
   ’Maybe the boy painted’
2. ? När efter lunchen | byggde | **jag** | ett litet fågelbord
   When after lunch$_{\text{DEF}}$ built I a little bird table
   ‘When after lunch did I build a little bird table’
3. Idag efter skolan | arbetade | **flickan** | hemma i köket
   Today after school$_{\text{DEF}}$ worked girl$_{\text{DEF}}$ home in kitchen$_{\text{DEF}}$
   ‘Today after school the girl worked in the kitchen’
4. Kanske | handlade | **han** | med Anders och Erik
   Maybe shopped he with Anders and Erik
   ‘Maybe he shopped with Anders and Erik’
5. Kanske | sov | **pojken**
   Maybe slept boy$_{\text{DEF}}$
   ’Maybe the boy slept’
6. **Pojken** | stod | nära trädet
   Boy$_{\text{DEF}}$ stood close tree$_{\text{DEF}}$
   ‘The boy stood close to the tree’
7. Idag | ringde | **hon** | till Sara
   Today called she to Sara
   ‘Today she called Sara’
8. Hemma | letade | **han** | upp alla pengarna
   At home looked he for all money$_{\text{DEF}}$
   ‘At home he looked for all the money’
9. **Jag** | åkte | inte | med flyg
   I went not by plane
   ‘I did not go by plane’
10. **Pojken** | gick | inte | hem till Anna
    Boy$_{\text{DEF}}$ went not home to Anna
    ‘The boy did not go home to Anna’
11. Naturligtvis | sov | **pojken**
    Of course slept boy$_{\text{DEF}}$
    ’Of course the boy slept’
12. ? När | kallade | **flickan**
    When called girl$_{\text{DEF}}$

’When did the girl call’

13. Här hos Anna | log | **jag** | under filten
    Here at Anna smiled I under blanket$_{\text{DEF}}$
    ‘Here at Anna’s I smiled under the blanket’
14. Självklart | åkte | **hon** | med Anders
    Obviously went he with Anders
    ‘Obviously he went with Anders’
15. Idag | målade | **pojken** | en bild av sjön
    Today painted boy$_{\text{DEF}}$ a picture of lake$_{\text{DEF}}$
    ‘Today the boy painted a picture of the lake’
16. ? När | slutade | **pojken** | att retas
    When stopped boy$_{\text{DEF}}$ to tease
    ‘When did the boy stop teasing’
17. Hemma i Lund | handlade | **han** | en bild på hunden
    At home in Lund bought he a picture of dog$_{\text{DEF}}$
    ‘ At home in Lund he bought a picture of the dog’
18. Hemma | målade | **pojken** | vackra blommor på en tavla
    At home painted boy$_{\text{DEF}}$ beautiful flowers on a painting
    ‘At home the boy painted beautiful flowers on a painting’
19. Idag efter lunchen | sparkade | **han** | boll
    Today after lunch$_{\text{DEF}}$ kicked he ball
    ‘Today after lunch he kicked the ball
20. **Hon** | såg | att Anna spelade fotboll
    She saw that Anna played football
    ‘She saw that Anna played football’
21. ? När | förlorade | **han** | matchen
    When lost he match$_{\text{DEF}}$
    ’When did he lose the match’
22. Naturligtvis | målade | **pojken** | kortet med blommorna till Anna
    Of course painted boy$_{\text{DEF}}$ card$_{\text{DEF}}$ with flowers$_{\text{DEF}}$ for Anna
    ‘Of course the boy painted the card with the flowers for Anna’
23. Hemma | tvättade | **hon** | filten
    At home washed she blanket$_{\text{DEF}}$
    ‘At home she washed the blanket’
24. Självklart | kom | **jag** | till Anna sent på kvällen
    Obviously came I to Anna late in evening$_{\text{DEF}}$
    ‘Obviously I came to Anna late in the evening’
25. Igår efter lunchen | slutade | **pojken**
    Yesterday after lunch$_{\text{DEF}}$ stopped boy$_{\text{DEF}}$
    ‘Yesterday after lunch the boy stopped’
26. ? När efter lunchen | orkade | **han** | springa upp för backen
    When after lunch$_{\text{DEF}}$ managed he run up hill$_{\text{DEF}}$
    ‘When after lunch did he manage to run up the hill’
27. **Pojken** | gick | nerför gatan
    Boy$_{\text{DEF}}$ went down street$_{\text{DEF}}$
    ’The boy went down the street’
28. Kanske | sprang | **pojken** | hem till Maria

Maybe ran boy$_{\text{DEF}}$ home to Maria
‘Mabye the boy ran home to Maria’
29. Idag på morgonen | handlade | **han** | alla de nybakade kakorna
Today in morning$_{\text{DEF}}$ bought he all the freshly baked cookies
‘Today in the morning he bought all the freshly baked cookies’
30. Naturligtvis | spelade | **pojken** | match
Of course played boy$_{\text{DEF}}$ match
‘Of course the boy played the match’
31. **Han** | förlorade | inte | en enda match
He lost not one single match
‘He did not lose one single match’
32. Naturligtvis | väntade | **han** | vid sin bänk
Of course waited he at his bench
‘Of course he waited at his bench’
33. **Jag** | flyttade | till Malmö
I moved to Malmö
’I moved to Malmö’
34. Naturligtvis | handlade | **han** | bollen till sina vänner
Of course bought he ball$_{\text{DEF}}$ for his friends
‘Of course he bought the ball for his friends’
35. **Han** | hörde | inte | vad hon sa
He heard not what she said
‘He did not hear what she said’
36. **Jag** | flyttade | inte | mina saker fast jag borde
I moved not my stuff even though I should
‘I did not move my stuff even though I should’
37. Idag efter skolan | sov | **pojken**
Today after school$_{\text{DEF}}$ slept boy$_{\text{DEF}}$
‘Today after school the boy slept’
38. Kanske | sparkade | **han** | fotboll på gräsmattan
Maybe kicked he football on lawn$_{\text{DEF}}$
‘Maybe he kicked the football on the lawn’
39. Naturligtvis | joggade | **pojken**
Of course jogged boy$_{\text{DEF}}$
’Of course the boy jogged’
40. **Han** | förlorade | spelet
He lost game$_{\text{DEF}}$
’He lost the game’
41. Idag efter lunchen | städade | **hon** | hela köket
Today after lunch$_{\text{DEF}}$ cleaned she whole kitchen$_{\text{DEF}}$
‘Today after lunch she cleaned the whole kitchen’
42. Förmodligen | kallade | **flickan** | högt
Probably called girl$_{\text{DEF}}$ loud
‘Probably the girl called out loud’
43. Hemma hos Erik | sov | **pojken** | i gästrummet
At home at Erik slept boy$_{\text{DEF}}$ in guest room$_{\text{DEF}}$
‘At Erik’s place the boy slept in the guest room’
44. ? När på eftermiddagen | gick | **pojken** | hem till Anna

When in afternoon$_{DEF}$ went boy$_{DEF}$ home to Anna
'When in the afternoon did the boy go home to Anna'

45. Här | <u>frös</u> | **jag** | på bänken
Here froze I on bench$_{DEF}$
'Here I was freezing on the bench'

46. Självklart | <u>byggde</u> | **pojken** | länge
Obviously built boy$_{DEF}$ long
'Obviously the boy built for a long time'

47. **Flickan** | <u>nickade</u> | inte | bollen i mål
Girl$_{DEF}$ nodded not ball$_{DEF}$ into goal
'The girl did not nod the ball into the goal'

48. ? När på morgonen | <u>bestämde</u> | **hon** | att det var hennes tur
When in morning$_{DEF}$ decided she that it was her turn
'When in the morning did she decide that it was her turn'

49. Kanske | <u>väntade</u> | **han** | länge
Maybe waited he long
'Maybe he waited for a long time'

50. Naturligtvis | <u>sprang</u> | **pojken** | med Sara i skogen
Of course ran boy$_{DEF}$ with Sara in forest$_{DEF}$
'Of course the boy ran with Sara into the forest'

51. Hemma | <u>ringde</u> | **hon** | till Erik
At home called she to Erik
'At home she called Erik'

52. ? När | <u>log</u> | **hon** | åt honom
When smiled she at him
'When did she smile at him'

53. ? När | <u>åkte</u> | **jag** | till Malmö
When went I to Malmö
'When did I go to Malmö'

54. Kanske | <u>joggade</u> | **pojken**
Maybe jogged boy$_{DEF}$
'Maybe the boy jogged'

55. Naturligtvis | <u>ramlade</u> | **han** | ner från trädet
Of course fell he down from tree$_{DEF}$
'Of course he fell from the tree'

56. Idag | <u>letade</u> | **han** | efter sin bok
Today looked he for his book
'Today he looked for his book'

57. **Hon** | <u>spelade</u> | fotboll
She played football
'She played football'

58. **Flickan** | <u>reste</u> | inte | med flyg utan med tåg
Girl$_{DEF}$ travelled not by plane but by train
'The girl did not travel by plane but by train'

59. Hemma | <u>läste</u> | **flickan** | tidningen ifred på sitt rum
At home read girl$_{DEF}$ newspaper$_{DEF}$ alone in her room
'At home the girl read the newspaper alone in her room'

60. Hemma hos Erik | arbetade | **pojken** | bra
At home at Erik worked boy$_{DEF}$ well
‘At Erik’s place the boy worked well’

61. Igår efter skolan | lovade | **han**
Yesterday after school$_{DEF}$ promised he
‘Yesterday after school he promised’

62. **Flickan** | nickade | bollen i mål
Girl$_{DEF}$ nodded ball$_{DEF}$ into goal
‘The girl nodded the ball into the goal’

63. Förmodligen | orkade | **han** | med sin fröken
Probably coped he with his teacher
‘Probably he coped with his teacher’

64. Igår | räknade | **hon** | alla de nybakade kakorna
Yesterday counted she all the freshly baked cookies
‘Yesterday she counted all the freshely baked cookies’

65. Naturligtvis | letade | **han** | medan Sara lekte
Of course searched he while Sara played
‘Of course he searched while Sara played’

66. **Jag** | stannade | inte | länge
I stayed not long
’I did not stay long’

67. **Han** | orkade | alla kakorna själv
He managed all cookies$_{DEF}$ himself
‘He managed all the cookies himself’

68. Idag | läste | **flickan** | vad som hade hänt
Today read girl$_{DEF}$ what that had happened
‘Today the girl read what had happened’

69. Naturligtvis | sparkade | **han** | boll med Erik och Anna
Of course kicked he ball with Eric and Anna
‘Of course he kicked the ball with Eric and Anna’

70. Hemma hos Erik | sparkade | **han** | på alla de vackra blommorna
At home at Erik kicked he at all the beautiful flowers
‘At Erik’s place he kicked at all the beautiful flowers’

71. **Jag** | lovade | inte | att hjälpa till
I promised not to help
‘I did not promise to help’

72. **Hon** | såg | inte | fåglarna
She saw not birds$_{DEF}$
’She did not see the birds’

73. Kanske | letade | **han** | medan hon städade
Maybe searched he while she cleaned
‘Maybe he searched while she cleaned’

74. Kanske | spelade | **pojken** | kort med farfar
Maybe played boy$_{DEF}$ cards with granddad
‘Maybe the boy played cards with granddad’

75. **Jag** | åkte | bil hela vägen
I drove car all way$_{DEF}$
‘I drove car all the way’

76. **Jag** | stannade | länge
I stayed long
‘I stayed for a long time’
77. Hemma hos Anna | städade | **hon** | undan sina kläder
At home at Anna cleaned she away her clothes
‘At Anna’s place she cleaned away her clothes’
78. Idag på morgonen | sprang | **pojken** | efter bollen nere vid sjön
Today in morning$_{DEF}$ ran boy$_{DEF}$ after ball$_{DEF}$ down by lake$_{DEF}$
‘Today in the morning the boy ran after the ball down by the lake’
79. Kanske | ramlade | **han** | hemma hos Anna
Maybe fell he at home at Anna
‘Maybe he fell at Anna’s place’
80. När på eftermiddagen | frös | **jag**
When in afternoon$_{DEF}$ froze I
’When in the afternoon was I freezing’
81. Självklart | bestämde | **han** | i köket
Obviously decided he in kitchen$_{DEF}$
‘Obviously he decided in the kitchen’
82. Hemma hos Anna | pratade | **flickan** | om Saras nya röda klänning
At home at Anna talked girl$_{DEF}$ about Sara’s new red dress
‘At Anna’s place the girl talked about Sara’s new red dress’
83. Hemma i Malmö | skrev | **hon** | tidningen
At home in Malmö wrote she newspaper$_{DEF}$
‘At home in Malmö she wrote the newspaper’
84. ? När | vågade | **flickan** | simma långt ut i sjön
When dared girl$_{DEF}$ swim far out into lake$_{DEF}$
‘When did the girl dare to swim far out into the lake’
85. Förmodligen | ropade | **jag** | på Sara som lekte
Probably shouted I at Sara who played
‘Probably I shouted at Sara who played’
86. Naturligtvis | städade | **hon** | hemma ikväll
Of course cleaned she at home tonight
‘Of course she cleaned at home tonight’
87. **Flickan** | kallade | på hunden
Girl$_{DEF}$ called at dog$_{DEF}$
’The girl called the dog’
88. Naturligtvis | arbetade | **pojken** | med sin fröken
Of course worked boy$_{DEF}$ with his teacher
‘Of course the boy worked with his teacher’
89. **Pojken** | slutade | inte | äta
Boy$_{DEF}$ stopped not eat
’The boy did not stop eating’
90. **Hon** | bestämde | hemma i sitt eget hus
She decided at home in her own house
‘She decided at home in her own house’
91. Idag | smakade | **hon** | Annas kakor
Today tasted she Anna’s cookies

‘Today she tasted Anna’s cookies’

92. Hemma | sjöng | **pojken** | tyst
At home sang boy$_{DEF}$ quietly
‘At home the boy sang quietly’

93. ? När | spelade | **hon** | fotboll med Erik
When played she football with Erik
‘When did she play football with Erik’

94. ? När på morgonen | kom | **jag** | hem
When in morning$_{DEF}$ came I home
‘When in the morning did I come home’

95. **Jag** | frös | inte | på morgonen
I froze not in morning$_{DEF}$
‘I was not freezing in the morning’

96. Idag efter skolan | väntade | **han** | hemma
Today after school$_{DEF}$ waited he at home
‘Today after school he waited at home’

97. **Flickan** | kallade | inte | på de andra barnen
Girl$_{DEF}$ called not at the other children
‘The girl did not call the other children’

98. Idag | sjöng | **pojken** | för Sara
Today sang boy$_{DEF}$ for Sara
‘Today the boy sang for Sara’

99. Förmodligen | stannade | **hon** | inne under den varma filten
Probably stayed she inside under the warm blanket
‘Probably she stayed under the warm blanket’

100. Naturligtvis | vilade | **han**
Of course rested he
’Of course he rested’

101. **Han** | ropade | på Anna
He shouted at Anna
’He shouted at Anna’

102. ? När | stod | **pojken** | stilla
When stood boy$_{DEF}$ still
’When did the boy stand still’

103. Kanske | vilade | **han** | på gräsmattan tillsammans med Anna
Maybe rested he on lawn$_{DEF}$ together with Anna
‘Maybe he rested on the lawn together with Anna’

104. ? När | ropade | **han** | på de andra barnen
When shouted he at the other children
‘When did he shout at the other children’

105. Hemma hos Anna | väntade | **han** | ensam
At home at Anna waited he alone
‘At Anna’s place he waited alone’

106. ? När på morgonen | stirrade | **pojken** | på väggen
When in morning$_{DEF}$ stared boy$_{DEF}$ at wall$_{DEF}$
‘When in the morning did the boy stare at the wall’

107. Kanske | arbetade | **flickan** | ensam i köket
Maybe worked girl$_{DEF}$ alone in kitchen$_{DEF}$

‘Maybe the girl worked alone in the kitchen’

108. Kanske | städade | **hon** | hela dagen
Maybe cleaned she all day$_{DEF}$
‘Maybe she cleaned all day’

109. Hemma i Malmö | sprang | **pojken** | till Annas hus
At home in Malmö ran boy$_{DEF}$ to Anna’s house
‘At home in Malmö the boy ran to Anna’s house’

110. **Jag** | lovade | att vara hemma i tid
I promised to be home in time
‘I promised to be home in time’

111. Självklart | nickade | **flickan** | till Erik senare på kvällen
Obviously nodded girl$_{DEF}$ to Erik later in evening$_{DEF}$
‘Obviously the girl nodded to Erik later in the evening$_{DEF}$’

112. **Hon** | log | inte | åt någon av pojkarna
She smiled not at any of boys$_{DEF}$
‘She did not smile at any of the boys’

113. Hemma | vilade | han | på bänken
At home rested he on bench$_{DEF}$
‘At home he rested on the bench’

114. Hemma | tystnade | hon
At home fell silent she
’At home she fell silent

115. **Flickan** | sålde | inte | kakorna
Girl$_{DEF}$ sold not cookies$_{DEF}$
’The girl did not sell the cookies’

116. Här hos Erik | flyttade | **hon** | undan sina kläder
Here at Erik moved she away her clothes
‘Here at Erik’s she moved away her clothes’

117. Naturligtvis | drack | **han** | mjölken
Of course drank she milk$_{DEF}$
‘Of course she drank the milk’

118. Idag | tvättade | **han** | kläderna$_{DEF}$
Today washed he clothes$_{DEF}$
’Today he washed the clothes’

119. **Jag** | byggde | ett litet hus
I built a little house
‘I built a little house’

120. Naturligtvis | sjöng | **pojken** | på matchen
Of course sang boy$_{DEF}$ at match$_{DEF}$
‘Of course the boy sang at the match’

121. Hemma | jobbade | **flickan**
At home worked girl$_{DEF}$
’At home the girl worked’

122. **Han** | hörde | vad som hade hänt
He heard what that had happened
‘He heard what had happened’

123. ? När efter skolan | reste | **flickan** | med tåget

When after school$_{\text{DEF}}$ travelled girl$_{\text{DEF}}$ by train$_{\text{DEF}}$
‘When after school did the girl travel by train’
124. Naturligtvis | berättade | **flickan** | att Erik var hungrig
Of course explained girl$_{\text{DEF}}$ that Erik was hungry
‘Of course the girl explained that Erik was hungry’
125. Naturligtvis | skrev | **hon**
Of course wrote she
’Of course she wrote’
126. Idag | jobbade | **flickan**
Today worked girl$_{\text{DEF}}$
’Today the girl worked’
127. **Jag** | frös | på eftermiddagen
I froze in afternoon$_{\text{DEF}}$
’I was freezing in the afternoon’
128. **Jag** | räknade | inte | allihop
I counted not everyone
’I did not count everyone’
129. Idag på eftermiddagen | skrev | **hon** | läxan
Today in afternoon$_{\text{DEF}}$ did she homework$_{\text{DEF}}$
‘Today in the afternoon she did the homework’
130. Här i Lund | vågade | **flickan**
Here in Lund dared girl$_{\text{DEF}}$
‘Here in Lund the girl dared’
131. Idag efter lunchen | pratade | **flickan** | bara med Eric och Anders
Today after lunch$_{\text{DEF}}$ talked girl$_{\text{DEF}}$ only to Eric and Anders
‘Today after lunch the girl talked only to Eric and Anders’
132. ? När efter skolan | hörde | **han** | hur de skrattat åt honom
When after school$_{\text{DEF}}$ heard he how they laughed at him
‘When after school did he hear how they laughed at him’
133. Igår efter skolan | stirrade | **jag** | efter bollen nere vid sjön
Yesterday after school$_{\text{DEF}}$ stared I at ball$_{\text{DEF}}$ down by lake$_{\text{DEF}}$
‘Yesterday after school I stared at the ball down by the lake’
134. Hemma | smakade | **hon** | godiset själv
At home tasted she candy$_{\text{DEF}}$ herself
‘At home she tested the candy herself’
135. Idag efter lunchen | spelade | **pojken** | på sitt piano
Today after lunch$_{\text{DEF}}$ played boy$_{\text{DEF}}$ on his piano
‘Today after lunch the boy played on his piano’
136. **Jag** | kom | inte | hem ensam
I came not home alone
‘I did not come home alone’
137. **Pojken** | stirrade | på Anna
Boy$_{\text{DEF}}$ stared at Anna
’The boy stared at Anna’
138. Igår | skrek | **jag** | hela vägen hem
Yesterday screamed I all way$_{\text{DEF}}$ home
‘Yesterday I screamed all the way home’
139. ? När | flyttade | **jag** | tillsammans med Anna

When moved I together with Anna
‘When did I move together with Anna’

140. Här | hörde | **han** | vad hon bakade hos Anna
Here heard he what he baked at Anna
‘Here he heard what he baked at Anna’s’

141. Hemma hos Anna | spelade | **pojken** | och läste tidningen
At home at Anna played boy$_{\text{DEF}}$ and read newspaper$_{\text{DEF}}$
‘At Anna’s place the boy played and read the newspaper’

142. Kanske | sjöng | **pojken** | med Anders
Maybe sang boy$_{\text{DEF}}$ with Anders
‘Maybe the boy sang with Anders’

143. **Jag** | räknade | pengarna
I counted money$_{\text{DEF}}$
’I counted the money’

144. Kanske | skrev | **hon** | snabbast
Maybe wrote she fastest
’Maybe she wrote fastest’

145. Kanske | kröp | **pojken**
Maybe crawled boy$_{\text{DEF}}$
’Maybe the boy crawled’

146. Idag på morgonen | skrattade | **flickan**
Today in morning$_{\text{DEF}}$ laughed girl$_{\text{DEF}}$
‘Today in the morning the girl laughed’

147. **Hon** | bestämde | inte
She decided not
’She did not decide’

148. Idag | vilade | **han** | hela dagen hemma i vardagsrummet
Today rested he all day$_{\text{DEF}}$ at home in living room$_{\text{DEF}}$
‘Today he rested all day at home in the living room’

149. Naturligtvis | tvättade | **hon**
Of course washed she
’Of course she washed’

150. Naturligtvis | kröp | **pojken**
Of course crawled boy$_{\text{DEF}}$
’Of course the boy crawled’

151. När efter lunchen | suckade | **hon**
When after lunch$_{\text{DEF}}$ seighed she
‘When after lunch did she seigh’

152. **Han** | ropade | inte | högre än någon annan
He shouted not louder than anybody else
‘He did not shout louder than anybody else’

153. **Flickan** | reste | med tåg till Anders
Girl$_{\text{DEF}}$ travelled by train to Anders
‘The girl travelled by train to Anders’

154. Förmodligen | pekade | **pojken** | i gräset
Probably pointed boy$_{\text{DEF}}$ to grass$_{\text{DEF}}$
‘Probably the boy pointed to the grass’

155. Kanske | berättade | **flickan** | högt
Maybe explained girl$_{DEF}$ loud
’Maybe the girl explained loud’
156. Kanske | drack | **han** | saft
Maybe drank he juice
’Maybe he drank juice’
157. **Pojken** | stirrade | inte
Boy$_{DEF}$ starred not
’The boy did not stare’
158. Naturligtvis | pratade | **flickan** | med Maria hemma i köket
Of course talked girl$_{DEF}$ to Maria at home in kitchen$_{DEF}$
‘Of course the girl talked to Maria at home in the kitchen’
159. Kanske | pratade | **flickan**
Maybe talked girl$_{DEF}$
’Maybe the girl talked’
160. Kanske | tvättade | **han** | när Sara gick på fest
Maybe washed he when Sara went to party
‘Maybe he washed when Sara went to the party’
161. Idag | låg | **flickan** | i gräset
Today lay girl$_{DEF}$ i grass$_{DEF}$
‘Today the girl lay in the grass’
162. Kanske | grät | **flickan** | under den varma filten
Maybe cried girl$_{DEF}$ under the warm blanket
‘Maybe the girl cried under the warm blanket’
163. ? När på morgonen | pekade | **jag** | mot fåglarna
When in morning$_{DEF}$ pointed I to birds$_{DEF}$
‘When in the morning did I point to the birds’
164. Här | stod | **jag** | under den varma filten
Here stood I under the warm blanket
‘Here I stood under the warm blanket’
165. Naturligtvis | vaknade | **hon** | under filten
Of course woke she under blanket$_{DEF}$
‘Of course she woke up under the blanket’
166. Hemma i Malmö | ramlade | **han** | bakom trädet
At home in Malmö fell he behind tree$_{DEF}$
‘At home in Malmö he fell behind the tree’
167. **Pojken** | vägrade | inte | att spela fotboll med Erik
Boy$_{DEF}$ refused not to play football with Erik
‘The boy did not refuse to play football with Erik’
168. Naturligtvis | låg | **flickan** | på soffan och tänkte
Of course lay girl$_{DEF}$ on sofa$_{DEF}$ and thought
‘Of course the girl lay on the sofa and thought’
169. Igår på eftermiddagen | sålde | **jag** | en bild av en sjö
Yesterday in afternoon$_{DEF}$ sold I a picture of a lake
‘Yesterday in the afternoon did I sold a picture of the lake’
170. ? När på eftermiddagen | sålde | **flickan** | kakorna
When in afternoon$_{DEF}$ sold girl$_{DEF}$ cookies$_{DEF}$
‘When in the afternoon did the girl sell the cookies’

171. Naturligtvis | jobbade | **flickan** | med läxan där hemma
Of course worked girl$_{\text{DEF}}$ on homework$_{\text{DEF}}$ there at home
‘Of course the girl worked on her homework at home’
172. Kanske | ringde | **hon** | till sin pappa
Maybe called she to her dad
‘Maybe she called her dad’
173. Kanske | smakade | **hon** | sin mat
Maybe tasted she her food
‘Maybe she tasted her food’
174. **Jag** | skrek | inte | åt någon av dem
I yelled not at anyone of them
‘I did not yell at anyone of them’
175. Idag på eftermiddagen | berättade | **flickan** | gärna
Today in afternoon$_{\text{DEF}}$ explained girl$_{\text{DEF}}$ gladly
‘Today in the afternoon the girl explained gladly’
176. Idag på morgonen | tittade | **hon** | på en bild av en sjö
Today in morning$_{\text{DEF}}$ looked she at a picture of a lake
‘Today in the morning she looked at a picture of a lake’
177. Naturligtvis | tystnade | **hon** | för att lyssna på fåglarna
Of course fell silent she in order to listen to birds$_{\text{DEF}}$
‘Of course she fell silent in order to listen to the birds’
178. **Pojken** | vägrade | ta på sig skjortan
Boy$_{\text{DEF}}$ refused put on himself shirt$_{\text{DEF}}$
‘The boy refused to put on the shirt’
179. Naturligtvis | tittade | **hon** | på henne i skoaffären
Of course looked she at her in shoe store$_{\text{DEF}}$
‘Of course she look at her in the shoe store’
180. Självklart | suckade | **jag** | och läste tidningen
Obviously seighed I and read newspaper$_{\text{DEF}}$
‘Obviously I seighed and read the newspaper’
181. **Jag** | pekade | inte | på honom
I pointed not at him
‘I did not point at him’
182. Hemma i Lund | joggade | **pojken** | till Anna sent på kvällen
At home in Lund jogged boy$_{\text{DEF}}$ to Anna late in evening$_{\text{DEF}}$
‘At home in Lund the boy jogged to Anna late in the evening’
183. Hemma i Malmö | skrattade | **flickan** | åt Eric när han busade
At home in Malmö laughed girl$_{\text{DEF}}$ about Eric when he joked
‘At home in Malmö the girl laughed about Eric when he was joking’
184. Idag på eftermiddagen | ramlade | **han** | och blev ledsen
Today in afternoon$_{\text{DEF}}$ fell he and got sad
‘Today in the afternoon he fell and got sad’
185. ? När | lyssnade | **han** | på sin fröken
When listened he to his teacher
‘When did he listen to his teacher’
186. **Flickan** | vågade | inte | ringa till Anders
Girl$_{\text{DEF}}$ dared not call to Anders

‘The girl did not dare to call Anders’

187. Hemma | vaknade | **hon** | i soffan
At home woke she on sofa$_{\text{DEF}}$
‘At home she woke up on the sofa’

188. Självklart | såg | **han** | hela köket
Obviously saw he whole kitchen$_{\text{DEF}}$
‘Obviously he saw the whole kitchen’

189. Kanske | jobbade | **flickan**
Maybe worked girl$_{\text{DEF}}$
’Maybe the girl worked’

190. **Han** | orkade | inte | springa snabbare än Erik
He managed not run faster than Erik
‘He did not manage to run faster than Erik’

191. ? När | räknade | **jag** | fel på ett prov
When counted I wrong at a test
‘When did I count wrong at a test’

192. ? När | nickade | **flickan** | mot Anders
When nodded girl$_{\text{DEF}}$ toward Anders
When did the girl nod towards Anders’

193. Hemma | drack | **han** | kaffe
At home drank he coffee
‘At home he drank coffee’

194. Idag på eftermiddagen | joggade | **pojken** | hela vägen hem
Today in afternoon$_{\text{DEF}}$ jogged boy$_{\text{DEF}}$ all way$_{\text{DEF}}$ home
‘Today in the afternoon the boy jogged all the way home’

195. Igår | spelade | **hon** | match
Yesterday played she match
’Yesterday she played the match’

196. ? När efter lunchen | skrek | **jag** | efter dig
When after lunch$_{\text{DEF}}$ yelled I at you
‘When after lunch did I yell at you’

197. Hemma | kröp | **pojken**
At home crawled boy$_{\text{DEF}}$
‘At home the boy crawled’

198. **Hon** | spelade | inte | fotboll med yngre barn
She played not football with younger kids
‘She did not play football with younger kids’

199. Förmodligen | lyssnade | jag | med Maria hemma i köket
Probably listened I with Maria at home in kitchen$_{\text{DEF}}$
‘Probably I listened with Maria at home in the kitchen’

200. ? När på eftermiddagen | såg | **hon** | de vackra blommorna
When in afternoon$_{\text{DEF}}$ saw she the beautiful flowers
‘When in the afternoon did she see the beautiful flowers’

201. Hemma i Lund | berättade | **flickan** | allt
At home in Lund told girl$_{\text{DEF}}$ everything
‘At home in Lund the girl told everything’

202. **Pojken** | slutade | att spela
Boy$_{\text{DEF}}$ stopped to play

’The boy stopped playing’

203. Hemma i Lund | tittade | **hon** | på Anna
At home in Lund looked she at Anna
‘At home in Lund she looked at Anna’

204. Här i Malmö | gick | **flickan** | på gräsmattan under trädet
Here in Malmö walked girl$_{DEF}$ on lawn$_{DEF}$ under tree$_{DEF}$
‘Here in Malmö the girl walked on the lawn under the tree’

205. Idag | grät | **flickan** | när hon såg hunden
Today cried girl$_{DEF}$ when she saw dog$_{DEF}$
‘Today the girl cried when she saw the dog’

206. **Jag** | pekade | på fåglarna i trädet
I pointed at birds$_{DEF}$ in tree$_{DEF}$
‘I pointed at the birds in the tree’

207. ? När efter lunchen | vägrade | **pojken** | att hjälpa till
When after lunch$_{DEF}$ refused boy$_{DEF}$ to help
‘When after lunch did the boy refuse to help’

208. Idag | drack | **han** | mjölk
Today drank he milk
’Today he drank milk’

209. Idag | tystnade | **hon** | snabbt
Today fell silent she fast
’Today she fell silent fast’

210. Naturligtvis | skrattade | **flickan**
Of course laughed girl$_{DEF}$
’Of course the girl laughed’

211. **Hon** | log | åt Erik och Anders
She smiled at Erik and Anders
‘She smiled at Erik and Anders’

212. Här | reste | **pojken** | till Sara
Here travelled boy$_{DEF}$ to Sara
‘Here the boy travelled to Sara’

213. Förmodligen | förlorade | **jag** | bollen
Probably lost I ball$_{DEF}$
’Probably I lost the ball’

214. Hemma | grät | **flickan** | på soffan hela kvällen
At home cried girl$_{DEF}$ on sofa$_{DEF}$ all evening$_{DEF}$
‘At home the girl cried on the sofa all evening’

215. **Hon** | suckade | inte | mer än Anders
She seighed not more than Anders
‘She did not seigh more than Anders’

216. ? När | stannade | **jag** | hemma
When stayed I home
’When did I stay home’

217. Kanske | låg | **flickan** | i soffan och läste
Maybe lay girl$_{DEF}$ on sofa$_{DEF}$ and read
Maybe the girl lay on the sofa and read

218. Hemma | låg | **flickan** | nedanför trädet och läste

At home lay girl$_{DEF}$ under tree$_{DEF}$ and read
'At home the girl lay under the tree and read'

219. Naturligtvis | <u>läste</u> | **flickan** | en bok för Sara
Of course read girl$_{DEF}$ a book to Sara
'Of course the girl read a book to Sara'

220. **Hon** | <u>suckade</u> | igen
He seighed again
'He seighed again'

221. **Pojken** | <u>stod</u> | inte | stilla en enda sekund
Boy$_{DEF}$ stood not still a single second
'The boy did not stand still a single second'

222. Kanske | <u>tittade</u> | **hon** | efter Sara som lekte
Maybe looked she for Sara who played
'Maybe she looked for Sara who played'

223. **Han** | <u>lyssnade</u> | inte | på vad Anna sa
He listened not to what Anna said
'He did not listen to what Anna said'

224. Idag | <u>vaknade</u> | **hon** | i köket
Today woke she in kitchen$_{DEF}$
'Today she woke up in the kitchen'

225. Kanske | <u>skrattade</u> | **flickan**
Maybe laughed girl$_{DEF}$
'Maybe the girl laughed'

226. Kanske | <u>tystnade</u> | **hon** | för att de var elaka
Maybe fell silent she because that they were mean
'Maybe she fell silent because they were mean'

227. **Jag** | <u>byggde</u> | inte | huset själv
I built not house$_{DEF}$ myself
'I did not build the house myself'

228. Naturligtvis | <u>smakade</u> | **hon** | vad hon bakade hos Anna
Of course tasted she what she baked at Anna
'Of course she tasted what she baked at Anna's'

229. Kanske | <u>vaknade</u> | **hon** | på gräsmattan under trädet
Maybe woke she on lawn$_{DEF}$ under tree$_{DEF}$
'Maybe she woke up on the lawn under the tree'

230. Naturligtvis | <u>ringde</u> | **hon** | till Erik senare på kvällen
Of course called she to Erik later in evening$_{DEF}$
'Of course she called Erik later in the evening'

231. ? När | <u>lovade</u> | **jag** | bort alla kakorna
When promised I away all cookies$_{DEF}$
'When did I promised away all the cookies'

232. **Han** | <u>lyssnade</u> | på alla fåglarna
He listened to all birds$_{DEF}$
'He listened to all the birds'

233. **Jag** | <u>kom</u> | så snabbt det gick
I came as fast it possible
'I came as fast as possible'

234. **Flickan** | <u>vågade</u> | klättra

Girl$_{DEF}$ dared not climb
’The girl did not dare to climb’

235. Naturligtvis | grät | **flickan** | på soffan
Of course cried girl$_{DEF}$ on sofa$_{DEF}$
‘Of course did the girl cry on the sofa’
236. Igår | vägrade | **flickan**
Yesterday refused girl$_{DEF}$
’Yesterday the girl refused’
237. **Jag** | skrek | åt dem allihop
I yelled at them all
‘I yelled at them all’
238. Kanske | läste | **flickan** | ensam hemma i köket
Maybe read girl$_{DEF}$ alone at home in kitchen$_{DEF}$
‘Maybe the girl read alone at home in the kitchen’
239. Idag | kröp | **pojken** | in under den varma filten
Today crawled boy$_{DEF}$ in under the warm covers
‘Today the boy crawled under the warm covers’
240. **Flickan** | sålde | alla sina leksaker
Girl$_{DEF}$ sold all her toys
‘The girl sold all her toys’

1.2 List 1 without fillers for the accuracy judgement task / ERP recordings

V3 sentences italicised, subjects in bold, verbs underlined for presentation purposes only

1. *Hemma **flickan** satt vid sin bänk.*
At home girl$_{DEF}$ sat at her desk.
‘At home the girl sat at her desk.’
2. Hemma lekte **hon** ensam i köket.
At home played she alone in kitchen$_{DEF}$.
‘At home she played alone in the kitchen.’
3. *Idag **hon** grät.*
Today she cried.
’Today she cried.’
4. *Idag **pojken** sprang hem till Maria.*
Today boy$_{DEF}$ run home to Maria.
‘Today the boy run home to Maria’
5. *Kanske **han** läste tidningen ifred på sitt rum.*
Maybe he read newspaper$_{DEF}$ alone in his room.
‘Maybe he read the newspaper alone in his room.’
6. Kanske handlade **flickan** en ny mössa.
Maybe bought girl$_{DEF}$ a new hat
‘Maybe the girl bought a new hat’

7. Hemma skrattade **pojken**.
At home laughed boy$_{\text{DEF}}$
’At home the boy laughed’
8. Kanske pratade **pojken** med Maria hemma i köket.
Maybe talked boy$_{\text{DEF}}$ to Maria at home in kitchen$_{\text{DEF}}$
‘Maybe the boy talked to Maria at home in the kitchen’
9. *Kanske **hon** tystnade.*
Maybe she fell silent
’Maybe she fell silent’
10. Idag tittade **flickan** på alla de vackra blommorna.
Today looked girl$_{\text{DEF}}$ at all the beautiful flowers
‘Today the girl looked at all the beautiful flowers’
11. Idag dansade **han** runt.
Today danced he around
’Today he danced around’
12. *Idag **hon** klättrade upp i trädet snabbt.*
Today she climbed up in tree$_{\text{DEF}}$ fast
‘Today she climbed up the tree fast’
13. Kanske tittade **hon** på Saras nya röda klänning.
Maybe looked she at Sara’s new red dress
‘Maybe she looked at Sara’s new red dress’
14. *Hemma **pojken** joggade.*
At home boy$_{\text{DEF}}$ jogged
’At home the boy jogged’
15. Idag vaknade **pojken** under filten.
Today woke boy$_{\text{DEF}}$ under blanket$_{\text{DEF}}$
‘Today the boy woke up under the blanket’
16. Hemma badade **han** gärna.
At home bathed he gladly
‘At home he bathed gladly’
17. *Idag **flickan** väntade på Anna.*
Today girl$_{\text{DEF}}$ waited for Anna
‘Today the girl waited for Anna’
18. *Kanske **han** jobbade med läxan där hemma.*
Maybe he worked on homework$_{\text{DEF}}$ there at home
‘Maybe he worked on his homework at home’
19. Kanske letade **pojken** efter Sara som lekte.
Maybe looked boy$_{\text{DEF}}$ for Sara who played
‘Maybe the boy looked for Sara who played’
20. *Kanske **flickan** joggade hem.*
Maybe girl$_{\text{DEF}}$ jogged home
’Maybe the girl jogged home’
21. Kanske dansade **hon** gärna.
Maybe danced she gladly
’Maybe she danced gladly’
22. Hemma tystnade **pojken** för att lyssna på fåglarna.
At home fell silent boy$_{\text{DEF}}$ to listen to birds$_{\text{DEF}}$
‘At home the boy fell silent to listen to the birds’

23. Idag läste **hon** tidningen.
    Today read she newspaper$_{DEF}$
    ’Today she read the newspaper’
24. Kanske väntade **han** på henne i skoaffären.
    Maybe waited he for her at shoe store$_{DEF}$
    ‘Maybe he waited for her at the shoe store’
25. *Hemma* ***han*** *tittade på Maria och log.*
    At home he looked at Maria and smiled
    ‘At home he looked at Maria and smiled’
26. Idag talade **flickan** med Eva och Maria.
    Today talked girl$_{DEF}$ to Eva and Maria
    ‘Today the girl talked to Eva and Maria’
27. Kanske lekte **han** ensam hemma i köket.
    Maybe played he alone at home in kitchen$_{DEF}$
    ‘Maybe he played alone at home in the kitchen’
28. *Hemma* ***pojken*** *letade efter sin bok.*
    At home boy$_{DEF}$ looked for his book
    ‘At home the boy looked for his book’
29. *Idag* ***han*** *jobbade gärna.*
    Today he worked gladly
    ’Today he worked gladly’
30. *Hemma* ***han*** *skrattade.*
    At home he laughed
    ’At home he laughed’
31. *Hemma* ***flickan*** *dansade hela dagen.*
    At home girl$_{DEF}$ danced all day$_{DEF}$
    ‘At home the girl danced all day’
32. Kanske betalade **hon**.
    Maybe paid she
    ’Maybe she paid’
33. Hemma ringde **han** och alla gick in.
    At home called he and all went in
    ‘At home he called and all went in’
34. *Kanske* ***pojken*** *kröp in under filten.*
    Maybe boy$_{DEF}$ crawled in under covers$_{DEF}$
    ‘Maybe the boy crawled under the covers’
35. Kanske jobbade **pojken** med läxan där hemma.
    Maybe worked boy$_{DEF}$ on homework$_{DEF}$ there at home
    ‘Maybe the boy worked on the homework there at home’
36. Idag tvättade **han**.
    Today washed he
    ’Today he washed’
37. *Hemma* ***han*** *sprang till Sara.*
    At home he ran to Sara
    ‘At home he ran to Sara’
38. Hemma smakade **pojken** alla de nybakade kakorna.
    At home tasted boy$_{DEF}$ all the freshly baked cookies

‘At home the boy tasted all the freshly baked cookies’

39. Idag betalade **han**.
Today paid he
’Today he paid’

40. Hemma talade **hon** med Erik.
At home talked she to Erik
‘At home she talked to Erik

41. Kanske talade **hon** med sin fröken.
Maybe talked she to her teacher
‘Maybe she talked to her teacher’

42. *Idag **pojken** betalade.*
Today boy$_{DEF}$ paid
’Today the boy paid’

43. Hemma ringde **pojken** och alla gick in.
At home called boy$_{DEF}$ and all went in
‘At home the boy called and all went in’

44. *Kanske **flickan** tittade på Saras nya röda klänning.*
Maybe girl$_{DEF}$ looked at Sara’s new red dress
‘Maybe the girl looked at Sara’s new red dress’

45. *Hemma **pojken** sov på soffan.*
At home boy$_{DEF}$ slept on sofa$_{DEF}$
‘At home the boy slept on the sofa’

46. Hemma drack **flickan** saft.
At home drank girl$_{DEF}$ juice
‘At home the girl drank juice’

47. *Idag **han** sprang hem till Maria.*
Today he ran home to Maria
‘Today he ran home to Maria’

48. *Hemma **flickan** sjöng medan hon städade.*
At home girl$_{DEF}$ sang while she cleaned
‘At home the girl sang while she cleaned’

49. Kanske satt **han** på soffan och tänkte.
Maybe sat he on sofa$_{DEF}$ and thought
‘Maybe he sat on the sofa and thought’

50. Kanske drack **han** mjölk.
Maybe drank he milk
’Maybe he drank milk’

51. Hemma ramlade **flickan**.
At home fell girl$_{DEF}$
’At home the girl fell’

52. Kanske sprang **flickan** snabbast.
Maybe ran girl$_{DEF}$ fastest
’Maybe the girl ran fastest’

53. *Hemma **han** skrev kortet med blommorna till Anna.*
At home he wrote card$_{DEF}$ with flowers$_{DEF}$ to Anna
‘At home he wrote the card with the flowers to Anna’

54. *Kanske **han** låg och läste tidningen.*
Maybe he lay and read newspaper$_{DEF}$

‘Maybe he lay and read the newspaper’

55. *Kanske* ***flickan*** *städade huset.*
    Maybe girl$_{\text{DEF}}$ cleaned house$_{\text{DEF}}$
    ’Maybe the girl cleaned the house’
56. *Idag* ***han*** *drack upp.*
    Today he drank up
    ’Today he drank up’
57. Kanske grät **flickan** för att de var elaka.
    Maybe cried girl$_{\text{DEF}}$ because that they were mean
    ‘Maybe the girl cried because they were mean’
58. Kanske hoppade **flickan**.
    Maybe jumped girl$_{\text{DEF}}$
    ’Maybe the girl jumped’
59. *Hemma* ***pojken*** *handlade upp alla pengarna.*
    At home boy$_{\text{DEF}}$ shopped for all money$_{\text{DEF}}$
    ‘At home the boy shopped for all his money’
60. *Kanske* ***hon*** *skrattade.*
    Maybe she laughed
    ’Maybe she laughed’
61. Kanske väntade **pojken** på henne i skoaffären.
    Maybe waited boy$_{\text{DEF}}$ for her inside shoe store$_{\text{DEF}}$
    ‘Maybe the boy waited for her inside the shoe store’
62. *Kanske* ***hon*** *grät för att de var elaka.*
    Maybe she cried because that they were mean
    ‘Maybe she cried because they were mean’
63. *Idag* ***han*** *ringde till sin pappa.*
    Today he called to his dad
    ‘Today he called his dad’
64. *Hemma* ***flickan*** *betalade för bollen.*
    At home girl$_{\text{DEF}}$ paid for ball$_{\text{DEF}}$
    ‘At home the girl paid for the ball’
65. *Hemma* ***pojken*** *hoppade högt.*
    At home boy$_{\text{DEF}}$ jumped high
    ‘At home the boy jumped high’
66. Idag joggade **flickan** hela vägen hem.
    Today jogged girl$_{\text{DEF}}$ all way$_{\text{DEF}}$ home
    ‘Today the girl jogged all the way home’
67. Idag berättade **hon** om sin nya mössa.
    Today told she about her new hat
    ‘Today she told about her new hat’
68. *Idag* ***flickan*** *grät.*
    Today girl$_{\text{DEF}}$ cried
    ’Today the girl cried’
69. Hemma svarade **pojken** på frågan.
    At home answered boy$_{\text{DEF}}$ on question
    ‘At home the boy answered the question’
70. Hemma sparkade **han** boll.

At home kicked he ball$_{DEF}$
‘At home he kicked the ball’

71. Kanske hoppade **hon**.
Maybe jumped she
’Maybe she jumped’

72. Hemma tvättade **hon** filten.
At home washed she blanket$_{DEF}$
‘At home she washed the blanket’

73. *Kanske* ***flickan*** *åt godiset själv.*
Maybe girl$_{DEF}$ ate candy$_{DEF}$ herself
‘Maybe the girl ate the candy herself’

74. *Kanske* ***pojken*** *drack mjölk.*
Maybe boy$_{DEF}$ drank milk
’Maybe the boy drank milk’

75. Kanske svarade **hon** att Erik var hungrig.
Maybe answered she that Erik was hungry
‘Maybe she answered that Erik was hungry’

76. *Hemma* ***hon*** *satt vid sin bänk.*
At home she sat at her desk
‘At home she sat at her desk’

77. *Idag* ***flickan*** *ramlade.*
Today girl$_{DEF}$ fell
’Today the girl fell’

78. Hemma betalade **hon** för bollen.
At home paid she for ball$_{DEF}$
‘At home she paid for the ball’

79. Idag ramlade **hon**.
Today fell she
’Today she fell’

80. Kanske städade **hon** huset.
Maybe cleaned she house$_{DEF}$
’Maybe she cleaned the house’

81. *Kanske* ***flickan*** *dansade bara med Eric och Anders.*
Maybe girl$_{DEF}$ danced only with Eric and Anders
‘Maybe the girl danced only with Eric and Anders’

82. *Kanske* ***hon*** *skrev i sin bok.*
Maybe she wrote in her book
‘Maybe she wrote in her book’

83. *Kanske* ***hon*** *vilade på gräsmattan.*
Maybe she rested on lawn$_{DEF}$
‘Maybe she rested on the lawn’

84. *Idag* ***flickan*** *badade.*
Today girl$_{DEF}$ bathed
’Today the girl bathed’

85. Kanske sprang **hon** snabbast.
Maybe ran she fastest
’Maybe she ran fastest’

86. Hemma arbetade **flickan** medan Sara lekte.

At home worked girl_{DEF} while Sara played
‘At home the girl worked while Sara played’

87. *Kanske* ***flickan*** *tystnade.*
Maybe girl DEF fell silent
’Maybe the girl fell silent’
88. *Idag* ***hon*** *talade med Eva och Maria.*
Today she talked to Eva and Maria
‘Today she talked to Eva and Maria’
89. *Hemma* ***han*** *tystnade för att lyssna på fåglarna.*
At home he fell silent for to listen to birds DEF
‘At home he fell silent to listen to the birds’
90. Hemma sov **han** på soffan.
At home slept he on sofa DEF
‘At home he slept on the sofa’
91. Idag målade **han** en bild av en sjö.
Today painted he a picture of a lake
‘Today he painted a picture of a lake’
92. Kanske lekte **pojken** ensam hemma i köket.
Maybe played boy DEF alone at home in kitchen DEF
‘Maybe the boy played alone at home in the kitchen’
93. Idag skrattade **flickan** åt Eric när han busade.
Today laughed girl DEF at Eric when he joked
‘Today the girl laughed at Eric when he joked’
94. *Hemma* ***han*** *åt godiset själv.*
At home he ate candy DEF himself
‘At home he ate the candy himself’
95. Hemma talade **flickan** med Erik.
At home talked girl DEF to Erik
‘At home the girl talked to Erik’
96. *Kanske* ***han*** *kröp in under filten.*
Maybe he crawled in under covers DEF
‘Maybe he crawled under the covers’
97. *Hemma* ***flickan*** *städade hela dagen.*
At home girl DEF cleaned all day DEF
‘At home the girl cleaned all day’
98. *Idag* ***hon*** *låg i soffan och läste.*
Today she lay on sofa and read
‘Today she lay on the sofa and read’
99. Idag läste **flickan** tidningen.
Today read girl DEF newspaper DEF
’Today the girl read the newspaper’
100. Idag letade **flickan** efter bollen nere vid sjön.
Today looked girl DEF for ball DEF down by lake DEF
‘Today the girl looked for the ball down by the lake’
101. *Kanske* ***flickan*** *skrev i sin bok.*
Maybe girl DEF wrote in her book
‘Maybe the girl wrote in her book’

102. Idag handlade **han** alla de nybakade kakorna.
Today bought he all the freshly baked cakes
‘Today he bought all the freshly baked cakes’
103. Kanske vaknade **hon**.
Maybe woke she
’Maybe she woke up’
104. *Kanske* ***pojken*** *ramlade och blev ledsen.*
Maybe boy$_{\text{DEF}}$ fell and got sad
‘Maybe the boy fell and got sad’
105. *Hemma* ***hon*** *sjöng medan hon städade.*
At home she sang while she cleaned
‘At home she sang while she cleaned’
106. *Idag* ***hon*** *väntade på Anna.*
Today she waited for Anna
‘Today she waited for Anna’
107. Kanske sjöng **han** för Sara.
Maybe sang he for Sara
‘Maybe he sang for Sara’
108. *Idag* ***hon*** *tittade på alla de vackra blommorna.*
Today she looked at all the beautiful flowers
‘Today she looked at all the beautiful flowers’
109. *Kanske* ***flickan*** *svarade att Erik var hungrig.*
Maybe girl$_{\text{DEF}}$ answered that Erik was hungry
‘Maybe the girl answered that Erik was hungry’
110. *Kanske* ***han*** *spelade kort med farfar.*
Maybe he played cards with granddad
‘Maybe he played cards with granddad’
111. Kanske handlade **hon** en ny mössa.
Maybe bought she a new hat
‘Maybe she bought a new hat’
112. Hemma badade **pojken** gärna.
At home bathed boy$_{\text{DEF}}$ gladly
‘At home the boy bathed gladly’
113. *Kanske* ***hon*** *smakade Annas kakor.*
Maybe she tasted Anna’s cookies
‘Maybe she tasted Anna’s cookies’
114. Idag **flickan** satt.
Today girl$_{\text{DEF}}$ sat
’Today the girl sat’
115. Kanske sparkade **han** boll.
Maybe kicked he ball
’Maybe he kicked the ball’
116. *Hemma* ***pojken*** *vaknade.*
At home boy$_{\text{DEF}}$ woke
’At home the boy woke up’
117. *Kanske* ***han*** *ramlade och blev ledsen.*
Maybe he fell and got sad
‘Maybe he fell and got sad’

118. Idag badade **hon**.
   Today bathed she
   'Today she bathed'
119. Kanske sjöng **pojken** för Sara.
   Maybe sang boy$_{\text{DEF}}$ for Sara
   'Maybe the boy sang for Sara'
120. *Kanske **hon** joggade hem.*
   Maybe he jogged home
   'Maybe he jogged home'
121. *Idag **hon** sjöng hemma i köket.*
   Today she sang at home in kitchen$_{\text{DEF}}$
   'Today she sang at home in the kitchen'
122. Idag klättrade **flickan** upp i trädet snabbt.
   Today climbed girl$_{\text{DEF}}$ up into tree$_{\text{DEF}}$ fast
   'Today the girl climbed up fast into the tree'
123. *Idag **flickan** åt sin mat.*
   Today girl$_{\text{DEF}}$ ate her food
   'Today the girl ate her food'
124. *Hemma **hon** grät.*
   At home she cried
   'At home she cried'
125. Kanske talade **flickan** med sin fröken.
   Maybe talked girl$_{\text{DEF}}$ to her teacher
   'Maybe the girl talked to her teacher'
126. *Idag **flickan** skrev i sin dagbok.*
   Today girl$_{\text{DEF}}$ wrote in her diary
   'Today the girl wrote in her diary'
127. Idag lekte **flickan** med Anders och Erik.
   Today played girl$_{\text{DEF}}$ with Anders and Erik
   'Today the girl played with Anders and Erik'
128. *Hemma **han** smakade alla de nybakade kakorna.*
   At home he tasted all the freshly baked cookies
   'At home he tasted all the freshly baked cookies'
129. Kanske skrattade **flickan**.
   Maybe laughed girl$_{\text{DEF}}$
   'Maybe the girl laughed'
130. Hemma målade **pojken** bänken.
   At home painted boy$_{\text{DEF}}$ bench$_{\text{DEF}}$
   'At home the boy painted the bench'
131. *Kanske **han** klättrade ner från trädet.*
   Maybe he climbed down from tree$_{\text{DEF}}$
   'Maybe he climbed down the tree'
132. Hemma skrev **pojken** kortet med blommorna till Anna.
   At home wrote boy$_{\text{DEF}}$ card$_{\text{DEF}}$ with flowers$_{\text{DEF}}$ to Anna
   'At home the boy wrote the card with flowers to Anna'
133. *Hemma **flickan** läste en bok för Sara.*
   At home girl$_{\text{DEF}}$ read a book to Sara

‘At home the girl read a book to Sara’

134. Idag tystnade **hon** när hon fick se hunden.
Today fell silent she when she got see dog$_{\text{DEF}}$
‘Today she fell silent when she got to see the dog’

135. Idag svarade **han** snabbt.
Today anwered he fast
’Today he answered fast’

136. Idag berättade **flickan** om sin nya mössa.
Today told girl$_{\text{DEF}}$ about her new hat
‘Today the girl told about her new hat’

137. *Idag* ***hon*** *åt sin mat.*
Today she ate her food
‘Today she ate her food’

138. *Idag* ***pojken*** *spelade fotboll på gräsmattan.*
Today boy$_{\text{DEF}}$ played football on lawn$_{\text{DEF}}$
‘Today the boy played football on the lawn’

139. Hemma städade hon hela dagen.
At home cleaned she all day$_{\text{DEF}}$
‘At home she cleaned all day’

140. *Idag* ***han*** *spelade fotboll på gräsmattan.*
Today he played football on lawn$_{\text{DEF}}$
‘Today he played football on the lawn’

141. *Hemma* ***han*** *jobbade gärna.*
At home he worked gladly
‘At home he worked gladly’

142. Hemma vilade **hon** länge.
At home rested she long
‘At home she rested for a long time’

143. Kanske tvättade **flickan** kläderna.
Maybe washed girl$_{\text{DEF}}$ clothes$_{\text{DEF}}$
’Maybe the girl washed the clothes’

144. Idag satt **hon**.
Today sat she
’Today she sat’

145. Kanske vilade **flickan** på gräsmattan.
Maybe rested girl$_{\text{DEF}}$ on lawn$_{\text{DEF}}$
‘Maybe the girl rested on the lawn’

146. *Idag* ***pojken*** *dansade runt.*
Today boy$_{\text{DEF}}$ danced around
’Today the boy danced around’

147. *Idag* ***han*** *arbetade.*
Today he worked
’Today he worked’

148. Idag handlade **pojken** alla de nybakade kakorna.
Today bought boy$_{\text{DEF}}$ all the freshly baked cookies
‘Today the boy bought all the freshly baked cookies’

149. Kanske smakade **flickan** Annas kakor.
Maybe tasted girl$_{\text{DEF}}$ Anna’s cookies

'Maybe the girl tasted Anna's cookies'

150. Hemma vilade **flickan** länge.
At home rested girl$_{DEF}$ long
'At home the girl rested long'

151. *Kanske* ***hon*** *målade en bild på hunden.*
Maybe she painted a picture of dog$_{DEF}$
'Maybe she painted a picture of the dog'

152. Kanske satt pojken på soffan och tänkte.
Maybe sat boy$_{DEF}$ on sofa$_{DEF}$ and thought
'Maybe the boy sat on the sofa and thought'

153. Kanske åt hon godiset själv.
Maybe ate she candy$_{DEF}$ herself
'Maybe she ate the candy herself'

154. *Kanske* ***pojken*** *ringde till Erik senare på kvällen.*
Maybe boy$_{DEF}$ called to Erik later in evening$_{DEF}$
'Maybe the boy called Erik later in the evening'

155. *Idag* ***pojken*** *jobbade gärna.*
Today boy$_{DEF}$ worked gladly
'Today the boy worked gladly'

156. *Idag* ***han*** *vaknade under filten.*
Today he woke under blanket$_{DEF}$
'Today he woke up under the blanket'

157. Hemma handlade **han** upp alla pengarna.
At home shopped he for all money$_{DEF}$
'At home he shopped for all the money'

158. Kanske vaknade **flickan**.
Maybe woke girl$_{DEF}$
'Maybe the girl woke up'

159. Hemma väntade **pojken** på att Anna skulle komma.
At home waited boy$_{DEF}$ for that Anna should come
'At home the boy waited for Anna to come'

160. *Hemma* ***han*** *joggade.*
At home he jogged
'At home he jogged'

161. Hemma dansade **hon** hela dagen.
At home danced she all day$_{DEF}$
'At home she danced all day'

162. *Hemma* ***flickan*** *grät.*
At home girl$_{DEF}$ cried
'At home the girl cried'

163. Idag pratade **hon** med sin fröken.
Today talked she to her teacher
'Today she talked to her teacher'

164. Idag städade **han** undan sina kläder.
Today cleaned he up his clothes$_{DEF}$
'Today he cleaned up his clothes'

165. Hemma tittade **pojken** på Maria och log.

At home looked boy$_{\text{DEF}}$ at Maria and smiled
‘At home the boy looked at Maria and smiled’

166. *Hemma **pojken** jobbade gärna.*
At home boy$_{\text{DEF}}$ worked gladly
‘At home the boy worked gladly’

167. *Idag **flickan** sparade pengarna till kakorna.*
Today girl$_{\text{DEF}}$ saved money$_{\text{DEF}}$ for cookies$_{\text{DEF}}$
‘Today the girl saved the money for the cookies’

168. Hemma åt **pojken** godiset själv.
At home ate boy$_{\text{DEF}}$ candy$_{\text{DEF}}$ himself
‘At home the boy ate the candy himself’

169. Hemma pratade **hon** med Eva och Maria.
At home talked she to Eva and Maria
‘At home she talked to Eva and Maria’

170. Hemma sprang **pojken** till Sara.
At home ran boy$_{\text{DEF}}$ to Sara
‘At home the boy ran to Sara’

171. *Idag **flickan** låg i soffan och läste.*
Today girl$_{\text{DEF}}$ lay on sofa$_{\text{DEF}}$ and read
‘Today the girl lay on the sofa and read’

172. Idag lekte **hon** med Anders och Erik.
Today played she with Anders and Erik
‘Today she played with Anders and Erik’

173. *Idag **pojken** drack upp.*
Today boy$_{\text{DEF}}$ drank up
’Today the boy drank up’

174. *Kanske **pojken** spelade kort med farfar.*
Maybe boy$_{\text{DEF}}$ played cards with granddad
‘Maybe the boy played cards with granddad’

175. *Kanske **hon** berättade vad som hade hänt.*
Maybe she told what that had happened
‘Maybe she told what had happened’

176. Idag sjöng **flickan** hemma i köket.
Today sang girl$_{\text{DEF}}$ at home in kitchen$_{\text{DEF}}$
‘Today the girl sang in the kitchen’

177. *Hemma **hon** drack saft.*
At home she drank juice
‘At home she drank juice’

178. *Hemma **hon** berättade vad hon bakade hos Anna.*
At home she told what she baked at Anna
‘At home she told what she had bake at Anna’s’

179. Hemma arbetade **hon** medan Sara lekte.
At home worked she while Sara played
‘At home she worked while Sara played’

180. Idag smakade **pojken** mjölken.
Today tasted boy$_{\text{DEF}}$ milk$_{\text{DEF}}$
’Today the boy tasted the milk’

181. *Kanske **pojken** sparkade boll.*

Maybe boy$_{DEF}$ kicked ball
’Maybe the boy kicked the ball’

182. Idag <u>ringde</u> **pojken** till sin pappa.
Today called boy$_{DEF}$ to his dad
‘Today the boy called his dad’

183. Kanske <u>betalade</u> **flickan**.
Maybe paid girl$_{DEF}$
’Maybe the girl paid’

184. *Hemma* ***pojken*** *<u>sparkade</u> boll.*
At home boy$_{DEF}$ kicked ball
‘At home the boy kicked the ball’

185. *Kanske* ***flickan*** *<u>berättade</u> vad som hade hänt.*
Maybe girl$_{DEF}$ told what that had happened
‘Maybe the girl told what had happened’

186. *Kanske* ***hon*** *<u>badade</u>.*
Maybe she bathed
’Maybe she bathed’

187. Kanske <u>sov</u> **hon** på soffan.
Maybe slept she on sofa$_{DEF}$
‘Maybe she slept on the sofa’

188. *Hemma* ***han*** *<u>målade</u> bänken.*
At home he painted bench$_{DEF}$
‘At home he painted the bench’

189. Hemma <u>klättrade</u> **pojken** upp till skåpen.
At home climbed boy$_{DEF}$ up to cupboard$_{DEF}$
‘At home the boy climbed up the cupboard’

190. *Idag* ***hon*** *<u>skrattade</u> åt Eric när han busade.*
Today she laughed about Eric when he joked
‘Today she laughed about Eric when he joked’

191. Kanske <u>låg</u> **pojken** och läste tidningen.
Maybe lay boy$_{DEF}$ and read newspaper$_{DEF}$
‘Maybe the boy lay and read the newspaper’

192. Idag <u>kröp</u> **flickan** upp i soffan till Anna.
Today crawled girl$_{DEF}$ up on sofa to Anna
‘Today the girl crawled up on the sofa to Anna’

193. *Kanske* ***pojken*** *<u>klättrade</u> ner från trädet.*
Maybe boy$_{DEF}$ climbed down from tree$_{DEF}$
‘Maybe the boy climbed down the tree’

194. *Kanske* ***flickan*** *<u>målade</u> en bild på hunden.*
Maybe girl$_{DEF}$ pinted a picture of dog$_{DEF}$
‘Maybe the girl painted a picture of the dog’

195. Kanske <u>badade</u> **flickan**.
Maybe bathed girl$_{DEF}$
’Maybe the girl bathed’

196. Idag <u>kröp</u> **hon** upp i soffan till Anna.
Today crawled she up on sofa$_{DEF}$ to Anna
‘Today she crawled up on the sofa to Anna’

197. *Idag* ***pojken*** *<u>hoppade</u> på studsmattan hela kvällen.*
Today boy$_{DEF}$ jumped on trampoline$_{DEF}$ all evening
'Today the boy jumped on the trampoline all evening'
198. *Hemma* ***flickan*** *<u>tvättade</u> filten.*
At home girl$_{DEF}$ washed blanket$_{DEF}$
'At home the girl washed the blanket'
199. *Idag* ***han*** *<u>hoppade</u> på studsmattan hela kvällen.*
Today he jumped on trampoline$_{DEF}$ all evening
'Today he jumped on the trampoline all evening'
200. *Kanske* ***flickan*** *<u>arbetade</u>.*
Maybe girl$_{DEF}$ worked
'Maybe the girl worked'
201. *Hemma* ***flickan*** *<u>pratade</u> med Eva och Maria.*
At home girl$_{DEF}$ talked to Eva and Maria
'At home the girl talked to Eva and Maria'
202. *Idag* ***hon*** *<u>joggade</u> hela vägen hem.*
Today she jogged all way$_{DEF}$ home
'Today she jogged all the way home'
203. *Idag* ***pojken*** *<u>målade</u> en bild av en sjö.*
Today boy$_{DEF}$ painted a picture of a lake
'Today the boy painted a picture of a lake'
204. *Idag* ***flickan*** *<u>vilade</u>.*
Today girl$_{DEF}$ rested
'Today the girl rested'
205. *Idag* ***hon*** *<u>letade</u> efter bollen nere vid sjön.*
Today she looked for ball$_{DEF}$ down by lake$_{DEF}$
'Today she looked for the ball down by the lake'
206. Idag <u>sparade</u> **hon** pengarna till kakorna.
Today saved she money$_{DEF}$ for cookies$_{DEF}$
'Today she saved the money for the cookies'
207. Kanske <u>tvättade</u> **hon** kläderna.
Maybe washed she clothes$_{DEF}$
'Maybe she washed the clothes'
208. Hemma <u>vaknade</u> **han**.
At home woke he
'At home he woke up'
209. *Hemma* ***flickan*** *<u>låg</u> på soffan och tänkte.*
At home girl$_{DEF}$ lay on sofa$_{DEF}$ and thought
'At home the girl lay on the sofa and thought'
210. Hemma <u>läste</u> **hon** en bok för Sara.
At home read she a book to Sara
'At home she read a book to Sara'
211. Idag <u>skrev</u> **hon** i sin dagbok.
Today wrote she in her diary
'Today she wrote in her diary'
212. *Idag* ***pojken*** *<u>städade</u> undan sina kläder.*
Today boy$_{DEF}$ cleaned up his clothes
'Today the boy cleaned up his clothes'

213. Hemma spelade **han** match.
At home played he match
'At home he played the match'
214. Hemma ramlade **hon**.
At home fell she
'At home she fell'
215. *Idag **flickan** pratade med sin fröken.*
Today girl$_{DEF}$ talked to her teacher
'Today the girl talked to her teacher'
216. *Hemma **han** kröp in under den varma filten.*
At home he crawled in under the warm covers$_{DEF}$
'At home he crawled under the warm covers'
217. Hemma letade **han** efter sin bok.
At home looked he for his book
'At home he looked for his book'
218. Idag arbetade **pojken**.
Today worked the boy$_{DEF}$
'Today the boy worked'
219. *Hemma **han** hoppade högt.*
At home he jumped high
'At home he jumped high'
220. Idag vilade **hon**.
Today rested she
'Today she rested'
221. Idag svarade **pojken** snabbt.
Today answered boy$_{DEF}$ fast
'Today the boy answered fast'
222. *Hemma **han** svarade på frågan.*
At home he answered on question
'At home he answered the question'
223. Hemma väntade **han** på att Anna skulle komma.
At home waited he for that Anna should come
'At home he waited for Anna to come'
224. *Kanske **han** arbetade.*
Maybe he worked
'Maybe he worked'
225. Idag tystnade **flickan** när hon fick se hunden.
Today fell silent girl$_{DEF}$ when she could see dog$_{DEF}$
'Today the girl fell silent when she could see the dog'
226. *Idag **han** sov i gräset.*
Today he slept on grass$_{DEF}$
'Today he slept on the grass'
227. *Hemma **hon** låg på soffan och tänkte.*
At home she lay on sofa$_{DEF}$ and thought
'At home she lay on the sofa and thought'
228. Idag tvättade **pojken**.
Today washed boy$_{DEF}$

’Today the boy washed’

229. *Hemma* ***pojken*** *spelade match.*
At home boy$_{DEF}$ played match
‘At home the boy played the match’

230. *Kanske* ***han*** *ringde till Erik senare på kvällen.*
Maybe he called to Erik later in evening$_{DEF}$
‘Maybe he called Erik later in the evening’

231. *Kanske* ***flickan*** *sov på soffan.*
Mabye girl$_{DEF}$ slept on sofa$_{DEF}$
’Maybe the girl slept on the sofa’

232. *Kanske* ***han*** *letade efter Sara som lekte.*
Maybe he looked for Sara who played
‘Maybe he looked for Sara who played’

233. *Kanske* ***pojken*** *läste tidningen ifred på sitt rum.*
Maybe boy$_{DEF}$ read newspaper$_{DEF}$ alone in his room
‘Maybe the boy read the newspaper alone in his room’

234. Kanske pratade **han** med Maria hemma i köket.
Maybe talked he to Maria at home in kitchen$_{DEF}$
‘Maybe he talked to Maria at home in the kitchen’

235. Idag smakade **han** mjölken.
Today tasted he milk$_{DEF}$
’Today he tasted the milk’

236. Idag sov **pojken** i gräset.
Today slept boy$_{DEF}$ on grass$_{DEF}$
‘Today the boy slept on the grass’

237. *Hemma* ***han*** *klättrade upp till skåpen.*
At home he climbed up the cupboard$_{DEF}$
‘At home he climbed up the cupboard’

238. Hemma kröp **pojken** in under den varma filten.
At home crawled boy$_{DEF}$ in under the warm covers
‘At home the boy crawled under the warm covers’

239. *Hemma* ***flickan*** *berättade vad hon bakade hos Anna.*
At home girl$_{DEF}$ told what she baked at Anna
‘At home the girl told what she had baked at Anna’s’

240. Hemma lekte **flickan** ensam i köket.
At home played girl$_{DEF}$ alone in kitchen$_{DEF}$
‘At home the girl played alone in the kitchen’
